# Dual transgene amelioration of Lama2-null muscular dystrophy

**DOI:** 10.1016/j.matbio.2023.03.001

**Published:** 2023-03-05

**Authors:** Karen K. McKee, Peter D. Yurchenco

**Affiliations:** a- Robert Wood Johnson Medical School, Rutgers University, Piscataway, NJ, USA

## Abstract

Null mutations of the *Lama2*-gene cause a severe congenital muscular dystrophy and associated neuropathy. In the absence of laminin-α2 (Lmα2) there is a compensatory replacement by Lmα4, a subunit that lacks the polymerization and α-dystroglycan (αDG)-binding properties of Lmα2. The dystrophic phenotype in the *dy*^*3K*^*/dy*^*3K*^ Lama2^−/−^ mouse were evaluated with transgenes driving expression of two synthetic laminin-binding linker proteins. Transgenic muscle-specific expression of αLNNd, a chimeric protein that enables α4-laminin polymerization, and miniagrin (*mag*), a protein that increases laminin binding to the receptor αDG, separately improved median mouse survival two-fold. The double transgenes (DT) improved mean survival three-fold with increases in overall body weight, muscle size, and grip strength, but, given absence of neuronal expression, did not prevent hindlimb paresis. Muscle improvements included increased myofiber size and number and reduced fibrosis. Myofiber hypertrophy with increased mTOR and Akt phosphorylation were characteristics of *mag*-*dy*^*3K*^*/dy*^*3K*^ and DT-*dy*^*3K*^*/dy*^*3K*^ muscle. Elevations of matrix-bound α4-, β1 and γ1 laminin subunits were detected in muscle extracts and immunostained sections in response to DT expression. Collectively, these findings reveal a complimentary polymerization and αDG-binding benefit to Lama2^−/−^ mouse muscle largely mediated through modified laminin-411.

## Introduction

Skeletal muscle myofibers are surrounded by a specialized extracellular matrix (ECM), the endomysial basement membrane (BM), supporting and protecting myofibers against mechanical forces generated by contraction and relaxation. Laminin-211 (α2β1γ1 heterotrimer, abbreviated Lm211), the major endomysial laminin, is important for BM assembly, architecture and receptor-interactions. Mutations within the *Lama2* gene coding for the Lmα2-subunit cause as much as 30% of cases of congenital muscular dystrophy (CMD). These mutations most commonly result in a complete or near-complete loss of protein subunit expression. Patients are floppy at birth, fail to ambulate, and often die of muscle wasting and respiratory failure by the second decade if untreated [1]. In a subset of cases, the subunit is defective and unable to participate in polymerization [2]. The resulting muscle pathology for both consists of myofiber degeneration and regeneration, chronic inflammation and progressive fibrosis [3].

While currently there is no known cure for the disease, several promising therapeutic candidates have emerged in recent years [4,5]. Of these, a number address repair of the structural defect that results from laminin mutations through alteration of gene expression. An approach from our work and those of colleagues focusses on modifying the compensatory laminin that is expressed in the dystrophic muscle sarcolemma, i.e. replacement of the α2 subunit by α4, mainly as Lm411 [4]. Lm411 cannot polymerize, binds weakly to integrins α6β1 and α7β1, and does not bind to αDG [6–8]. Normally confined to vessels, peripheral nerve branches and neuromuscular junctions in muscle, α4-laminin has been found to contribute to vasculature maturation and to myelination [9–11] in which the underlying mechanisms are thought to involve receptor interactions in the absence of polymerization. One possible contribution is that Lm411 normally competes for the α2-LN domains bound to the β1-γ1 LN dimers in polymers of Lm211, reducing signaling through modulation of extracellular matrix layer stiffness [12]. A balance of polymerizing (Lm211) and non-polymerizing (Lm411) laminins may be required for optimal function. In this study, laminin-binding linker proteins were used to alter α4-laminin such that activities present in α2-laminin were transferred to α4-laminins [8].

The muscle sarcolemmal BM can be described as a layer of transversely linked proteins extending from stromal-interface collagen-VI to BM to trans-membrane integrin α7β1 and α/β-dystroglycan complex to cytoskeletal dystrophin. The BM in turn is connected by inter-laminin-211 and inter-collagen-IV bonds that form laterally-oriented planar polymer arrays [13]. This structural arrangement helps to explain how mutations that alter these components result in muscular dystrophies ranging in severity from mild to severe [3]. A key step in the assembly of the BM is provided by the Lm211 attachment to αDG and α7β1 integrin receptors and Lm211 self-assembly. αDG is thought to provide the more critical receptor link in muscle [14]. Several mutations that alter the αDG mannosyl-O-linked carbohydrate (matriglycan) essential for ligand binding by Lm-211, agrin and perlecan cause severe congenital and limb girdle dystrophies. In contrast, mutations ablating α7β1 integrin function result in a milder dystrophy [14]. The importance of the lateralizing links provided by laminin polymers is illustrated by the dystrophy that develops with mutations in the LN domain selectively preventing laminin polymerization [2,15,16].

**Fig. 1.**
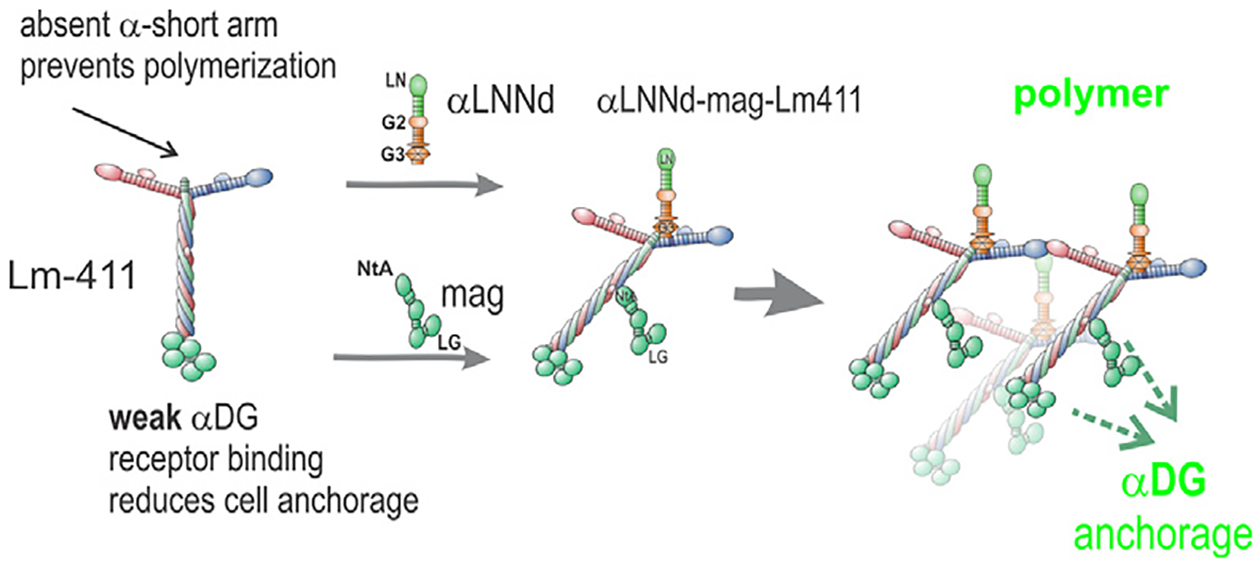
αLNNd and mag alteration of Lm411 functions. αLNNd, a chimeric protein consisting of Lmα1LN-LEa fused N-terminally to nidogen-1 G2-G3 domains. The LN polymerization domain provides the missing αLN domain of Lm411 while the G3 domain binds αLNNd to the Lmγ1 LEb3 domain, creating an artificial short arm. Domain G2 binds to collagen-IV and perlecan. Miniagrin (mag) consists of the laminin-binding NtA domain and first follistatin domain fused to the agrin C-terminal LG and LE domains. The LG domains bind to the α-dystroglycan receptor. Dual modification of Lm411 allows it to polymerize and bind to a cell surface receptor.

A chimeric protein, αLNNd, binds to laminins at the nidogen-binding locus in the Lmγ1 short arm and enables polymerization of laminins with an absent or defective N-terminal αLN domain [2,17] (Fig. 1). The *dy*^*2J*^*/dy*^*2J*^ mouse, a model for a less severe form of laminin-deficiency, expresses non-polymerizing α2-laminin resulting from a destabilizing in-frame deletion within the α2LN domain [15]. Transgenic muscle expression of αLNNd in *dy*^*2J*^*/dy*^*2J*^ dystrophic mice was found to enable polymerization of the defective Lm211 and substantially improved mouse muscle strength and histology [18]. A second engineered laminin-binding protein is mini-agrin (*mag*). Consisting of an internally shortened version of non-neuronal agrin, it binds to the laminin coiled-coil and the αDG receptor [8,19]. Transgenic muscle-specific expression of this protein in the *dy*^*W*^*/dy*^*W*^ Lama2 hypomorphic mouse [20] with considerably reduced Lmα2 subunit expression partially restored muscle function and histology [19]. In contrast, co-expression of both proteins in the *dy*^*W*^*/dy*^*W*^ hypomorphic mouse improved histology, survival, body weights and strength to a greater extent than either transgene alone [8].

In the current study, we evaluated dual transgenic expression of the two linker proteins in the full knockout of the Lama2 gene (*dy*^*3K*^*/dy*^*3K*^, [21]). We focused on *dy*^*3K*^*/dy*^*3K*^ because, unlike *dy*^*W*^*/dy*^*W*^, there is no detectable expression of surviving truncated α2 subunits lacking LN domains [20] to complicate analysis, muscle laminin levels are challenging low, and the mouse model corresponds to the most common presentation of Lama2-RD [22–24]. The mice exhibit the severest dystrophic phenotype of all Lama2 dystrophic models [25]. We report that each transgene provided similar limited amelioration with considerable benefit achieved with double transgene expression as measured by mouse survival, weights, muscle size, and grip strength. Extraction studies revealed that an increased amount of laminin, mostly Lm411, was retained in extracellular matrix in the presence of linker proteins.

## Results

Predictions were that the extent of Lm411 assembly on myofiber surfaces depends on protein concentration with the most efficient assembly occurring when both polymerization and anchorage are facilitated. Since laminins have only a single high-affinity binding site for the nidogen G3 domain (γ1LEb3) [26] and a single high-affinity binding site for the agrin NtA domain (coiled-coil) [27], the three proteins should assemble on cells as ternary complexes. When cultured myotubes were incubated with equimolar Lm411, αLNNd and *mag*, cell surface assembly (measured as accumulation) was found to be concentration dependent with the greatest accumulation seen with the combination of linker proteins, supporting the expectations (Suppl. Fig. S1).

### Transgene expression improves mouse survival, growth and muscle function:

**Fig. 2.**
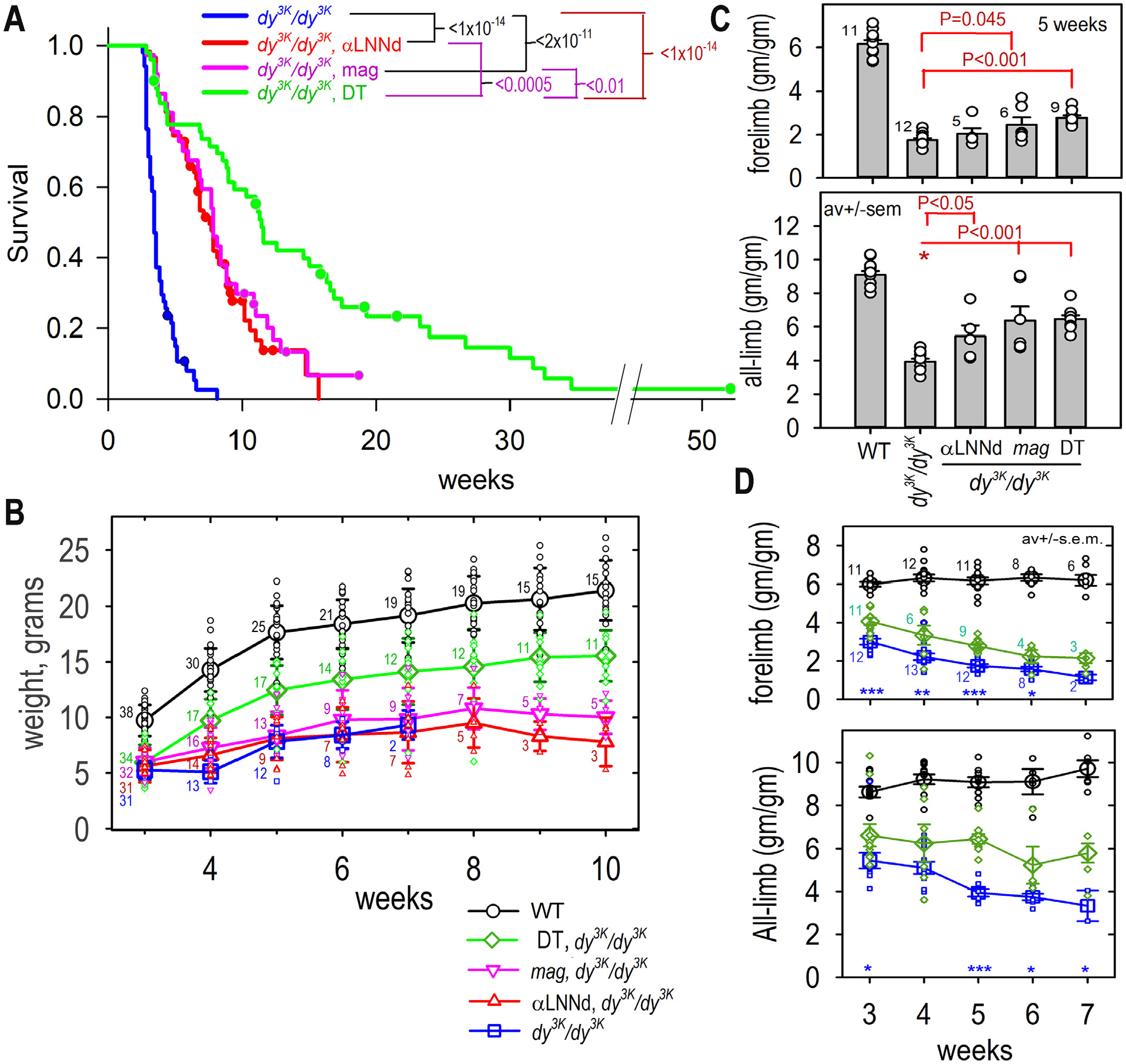
Transgenic muscle expression of linker proteins improvement of survival, weight gain and grip endurance. A. *Mouse survival*. Kaplan-Meier survival with log-rank analysis. Censored data (following approved humane guidelines) for sacrificed mice indicated with dots. Log-rank probabilities comparing the different mouse groups are shown above graph (from 50 *dy*^*3K*^*/dy*^*3K*^, 58 αLNNd-*dy*^*3K*^*/dy*^*3K*^. 36 *mag*-*dy*^*3K*^*/dy*^*3K*^, and 49 DT-*dy*^*3K*^*/dy*^*3K*^ mice). B. *Mouse weights*. WT, dystrophic and dystrophic mice expression one or two transgenes were weighed at the indicated times (av. ± s.d. with individual values (open symbols) shown; numbers of mice indicated with small numerals for each averaged data point). C. *Specific grip strength, 5 weeks*. Comparison of WT, *dy*^*3K*^*/dy*^*3K*^ without/with one or both transgenes (av. ± s.e.m. with individual values (open circles) shown. D. *Specific grip strength at different ages*. Specific grip strengths of DT-*dy*^*3K*^*/dy*^*3K*^, WT and *dy*^*3K*^*/dy*^*3K*^ mice at different ages (av. ± s.e.m. with individual values (open circles): ***, P<0.001; **, P<0.01; *, P<0.01 for *dy*^*3K*^*/dy*^*3K*^ cf. DT-*dy*^*3K*^*/dy*^*3K*^). P values determined by 1-way ANOVA followed by pairwise Holm-Sidak test (mouse numbers indicated with numerals).

αLNNd and *mag* transgenes, both driven by the muscle creatine kinase (MCK) promoter, were prepared and evaluated for sarcolemmal expression in muscle as previously described [18,19]. The transgenic mice were bred with *dy*^*3K*^/+ mice to generate wild-type (+/+, *dy*^*3K*^/+ without or with transgenes), *dy*^*3K*^*/dy*^*3K*^ dystrophic, and *dy*^*3K*^*/dy*^*3K*^ mice expressing one or both transgenes. Survival times, weights and specific grip strengths were compared among dystrophic mice without and with single and double transgene expression (Fig. 2).

The dystrophic *dy*^*3K*^*/dy*^*3K*^ mice were smaller than WT mice, developing characteristic hindlimb behavior with a slowly progressive gait abnormality by about two weeks of age. Upon picking up the mice by their tails at weaning, normal mice extended their hindlimbs while dystrophic mice variably flexed their hindlimbs, bringing them close against the abdomen. The DT-*dy*^*3K*^*/dy*^*3K*^ mouse gait abnormality was largely confined to the hindlimbs, initially without greatly affecting mobility (Supplemental Figure S3 and Video). The *dy*^*3K*^*/dy*^*3K*^ mice, in the absence of transgenes, were smaller and exhibited limited mobility. They usually died before development of obvious hindlimb contractures. The mice bearing transgenes gradually developed hindlimb extension contractures over the ensuing weeks, initially transiently. Since these anomalies have been reported in Schwann cell-specific laminin-γ1 deficient mice, it is likely that these particular behavioral defects are due to sciatic nerve amyelination rather than a primary muscle defect [11,28,29]. Single and double transgenes improved survival and weights; however, the hindlimb abnormalities were not ameliorated by the muscle-specific transgenes.

### Survival gains in the dy^3K^/dy^3K^ mouse:

The survival of the four groups of dystrophic mice (*dy*^*3K*^*/dy*^*3K*^, αLNNd-*dy*^*3K*^*/dy*^*3K*^*, mag*-*dy*^*3K*^*/dy*^*3K*^, and DT-*dy*^*3K*^*/dy*^*3K*^) was examined and plotted by the Kaplan-Meir method with log-rank analysis (Fig. 2A). Those mice approaching a moribund state were sacrificed according to an approved protocol. Mice sacrificed for this reason or for tissue retrieval are indicated as censored data points along with mice sacrificed for tissue retrieval. The *dy*^*3K*^*/dy*^*3K*^ mice without transgene were short-lived with a mean survival of 4 weeks and no survivors beyond 8 weeks of age. The *dy*^*3K*^*/dy*^*3K*^ mice with single transgene expression survived about twice as long with no statistical difference between αLNNd- *dy*^*3K*^*/dy*^*3K*^ and *mag*-*dy*^*3K*^*/dy*^*3K*^. The *dy*^*3K*^*/dy*^*3K*^ mice with double-transgene expression lived the longest with a three-fold mean increase. One mouse survived longer than one year. No significant survival sex difference was noted (Suppl. Fig. S2).

### Weight gain:

The WT mice doubled their weights between 3 and 10 weeks of age while the *dy*^*3K*^*/dy*^*3K*^ mice without transgene maintained their wean weight until death (Fig. 2B). In comparison, homozygous dystrophic mice expressing either the αLNNd or *mag* transgene alone exhibited little increase in weight, while those expressing both transgenes increased considerably higher than that seen with single transgene, but still below that of WT mice.

### Muscle function:

Forelimb and all-limb specific muscle grip strength was evaluated (Fig. 2C,D). Comparisons made among all genotypes at 5 weeks age revealed increased grip strength for DT-*dy*^*3K*^*/dy*^*3K*^ mice compared to *dy*^*3K*^*/dy*^*3K*^ with small differences among single transgene and DT mice. Measurements were compared among DT- *dy*^*3K*^*/dy*^*3K*^, WT and *dy*^*3K*^*/dy*^*3K*^ mice over a four-week period – thereafter, the hindlimb paresis and associated progressive extensor hindlimb contractures interfered with all-limb measurements and surviving *dy*^*3K*^*/dy*^*3K*^ were no longer available for comparison. Significant but small (forelimb) to modest (all-limb) improvements of both grip strength were observed for DT-*dy3*^*K*^*/dy*^*3K*^ compared to *dy*^*3K*^*/dy*^*3K*^ mice.

### Muscle histology:

**Fig. 3.**
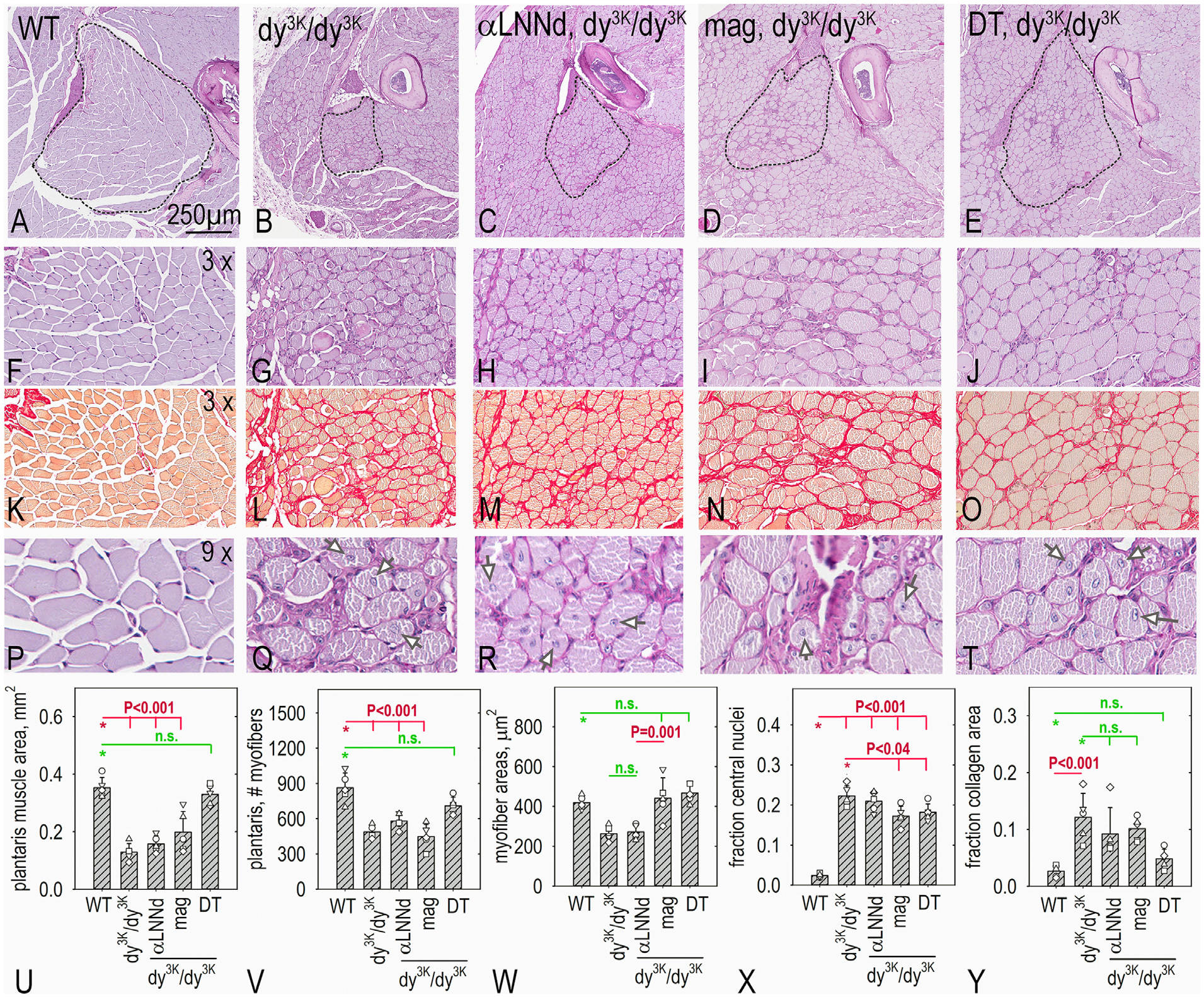
Histopathology of plantaris. Muscle from WT, *dy*^*3K*^*/dy*^*3K*^, αLNNd *dy*^*3K*^*/dy*^*3K*^*, mag, dy*^*3K*^*/dy*^*3K*^ and double-trans-gene (DT), dy^3K^/dy^3K^ mice at 6 weeks of age (n=5 mice for each), were analyzed after staining with PAS (panels A-J, P-T) and Picro-Sirius red (panels K-O). Av. ± s.d., with individual mouse values (open symbols) shown (panels U-Y) with representative images shown. P values determined by 1-way ANOVA followed by pairwise Holm-Sidak test. Overall muscle size and myofiber count were greatly reduced in *dy*^*3K*^*/dy*^*3K*^ and considerably increased in DT-treated dy^3K^/dy^3K^ mice. Average *mag-dy*^*3K*^*/dy*^*3K*^ myofiber cross-sectional areas were significantly greater than those αLNNd-*dy*^*3K*^*/dy*^*3K*^ myofibers. Collagen, measured as an area fraction, was slightly reduced with each transgene alone and reduced to levels approaching those of WT in DT- *dy*^*3K*^*/dy*^*3K*^ mice. The fraction of myofibers with central nuclei was similarly elevated in all dystrophic mice (arrows in P-T show examples).

**Fig. 4.**
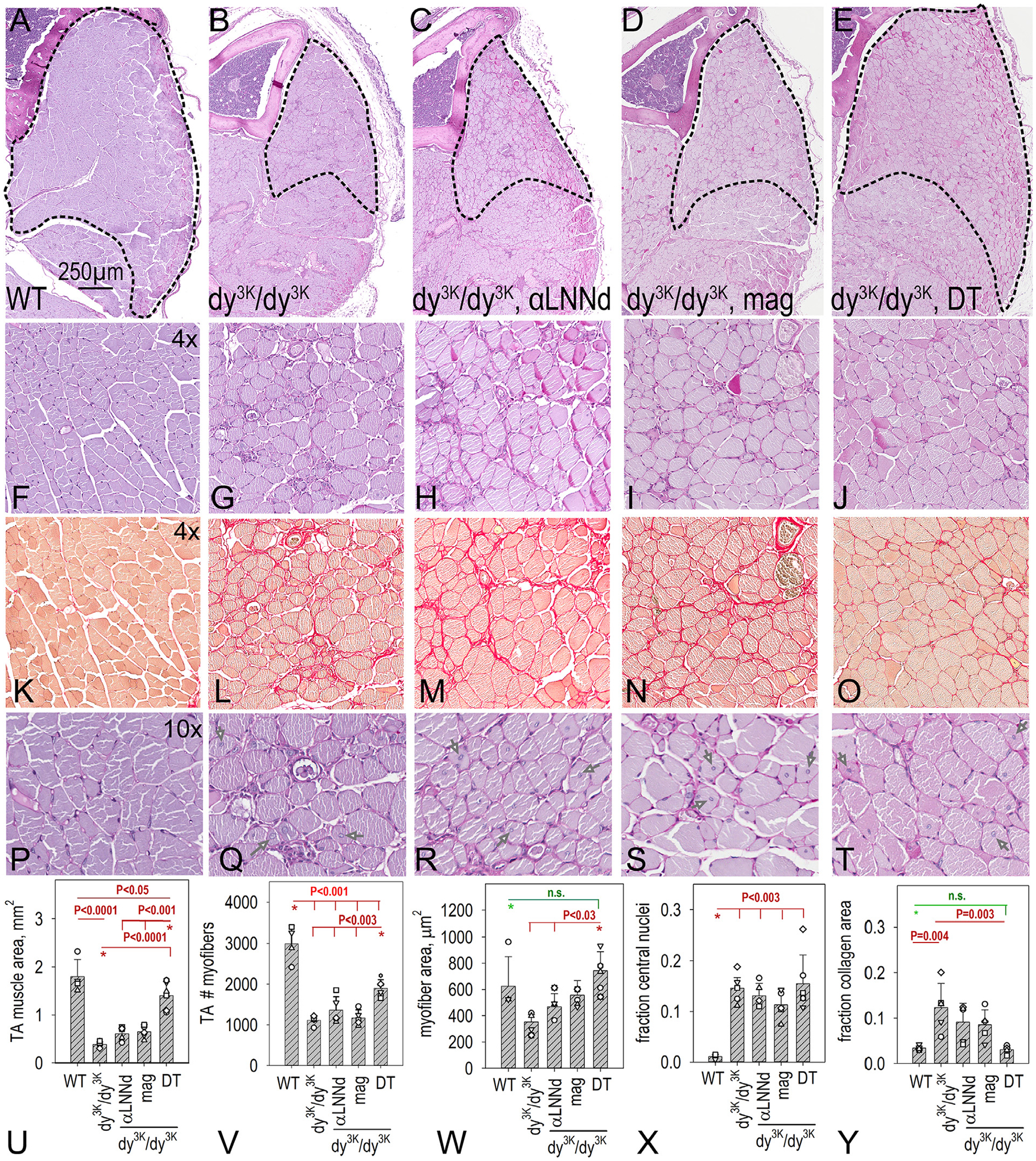
Histopathology of tibialis anterior. Muscle from WT (n=4), *dy*^*3K*^*/dy*^*3K*^ (n=5), αLNNd *dy*^*3K*^*/dy*^*3K*^ (n=5), *mag*-*dy*^*3K*^*/dy*^*3K*^ (n=5) and DT-*dy*^*3K*^*/dy*^*3K*^ (n=6) mice at 6 weeks of age were analyzed after staining with PAS (panels A-J) and picro-Sirius red (panels K-O). Av. ± s.d., with individual mouse values (open symbols) shown (panels P-T, arrows in P-T show examples). P values determined by 1-way ANOVA followed by pairwise Holm-Sidak test. Overall muscle size, myofiber count, and summed myofiber areas were reduced in *dy*^*3K*^*/dy*^*3K*^, slightly or not increased with each transgene alone, and considerably increased in DT- *dy*^*3K*^*/dy*^*3K*^ mice. Collagen, measured as area fraction, was slightly reduced with each transgene alone and reduced to levels approaching those of WT in DT- *dy*^*3K*^*/dy*^*3K*^ mice. The fraction of myofibers with central nuclei were elevated in all dystrophic mice.

Hindlimb skeletal muscles was examined histologically in mid-length cross-sections of plantaris (Fig. 3), tibialis anterior (Fig. 4) and triceps (Suppl. Fig. S5) with morphometric measurements. Periodic acid Schiff (PAS) was used to assess myofiber size and shape and delineate the sarcolemmal BMs while Picro-Sirius Red (PSR) was used to characterize collagen accumulation (fibrosis). Dystrophic changes were more severe in plantaris compared to tibialis anaterior (TA). The number of fibers in plantaris and TA was reduced in *dy*^*3K*^*/dy*^*3K*^ in which many of the myofibers were of small caliber. Expression of a single transgene, whether αLNNd or *mag*, was associated with limited change in plantaris or TA myofiber counts. In contrast to αLNNd-treatment, an increase in average individual myofiber caliber in *mag*-*dy*^*3K*^*/dy*^*3K*^ mice was noted in plantaris (as well as hindlimb gastrocnemius and rectus femoris), but not TA. A significant increase in the number of myofibers/muscle in plantaris and TA and the average myofiber area was noted in DT-*dy*^*3K*^*/dy*^*3K*^. The fraction of myofibers with non-peripheral (“central”) nuclei, a marker for regeneration, was high in all *dy*^*3K*^*/dy*^*3K*^ muscle, regardless of transgene expression. This is in contrast with αLNNd transgene expression in *dy*^*2J*^*/dy*^*2J*^ muscle but similar to that observed with double transgene *dy*^*W*^*/dy*^*W*^ muscle [8,18] and older *dy*^*3K*^*/dy*^*3K*^ mice rescued with a full-length Lmα1 transgene [30]. Collagen increases were noted in *dy*^*3K*^*/dy*^*3K*^ muscles in a largely peri-sarcolemmal distribution. This was somewhat reduced in dystrophic muscle with single transgene expression, and considerably reduced with DT expression. Immunostaining of macrophages in muscle (3 weeks age) for F4/80 (reflecting chronic inflammation) revealed decreased staining in the presence of both transgenes (Suppl. Fig. S6).

### Muscle hypertrophy and mTOR/Akt phosphorylation.

**Fig. 5.**
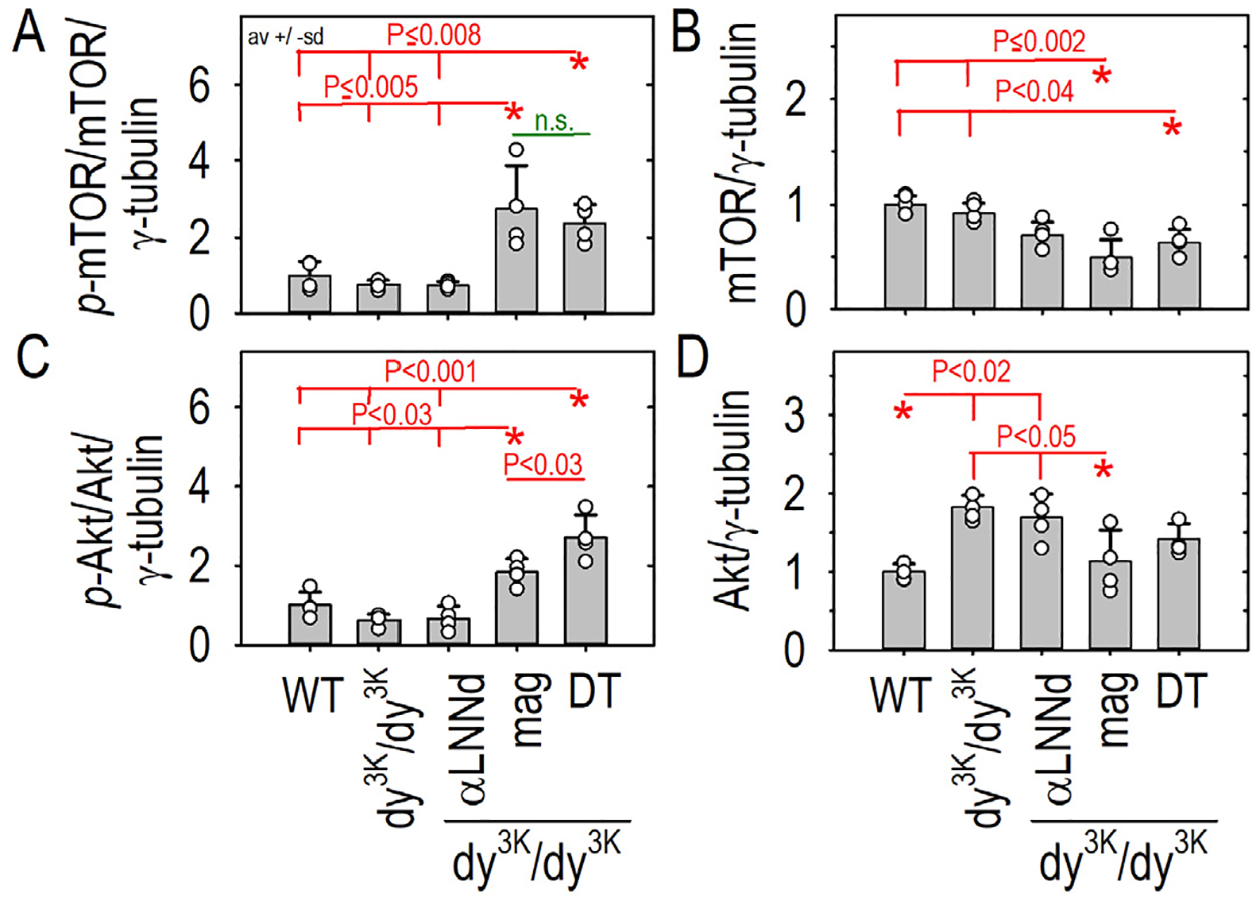
mTOR and Akt phosphorylation increases in response to miniagrin expression. Forelimb triceps muscle was obtained from four each of WT and dystrophic (*dy*^*3K*^*/dy*^*3K*^) mice without and with transgene expression at six weeks of age. Muscle was ground and extracted with Triton buffer and analyzed by reducing SDS-PAGE (7.5%) with detection of phosphorylated (A,C) and total (B,D) mTOR and Akt. Av. ± s.d with individual mouse values (open symbols) shown. P values determined by 1-way ANOVA followed by pairwise Holm-Sidak test. Phospho-mTOR and phosphor-Akt were elevated in *mag* and double transgene expressing dystrophic muscle. Changes in total mTOR and Akt were also detected.

Further examination of plantaris (Fig. 3) and other severely affected muscles (by inspection) revealed that *mag*- *dy*^*3K*^*/dy*^*3K*^ myofibers were hyper-trophic (i.e. larger cross-sectional areas) compared to those of αLNNd- *dy*^*3K*^*/dy*^*3K*^ and untreated *dy*^*3K*^*/dy*^*3K*^ myofibers. The mammalian target of rapamycin (mTOR), a regulator of protein translation, has been implicated in the control of muscle mass such that inactivation of mTOR results in atrophy [31]. Phosphorylation of mTOR and Akt was examined in muscle of WT, *dy*^*3K*^*/dy*^*3K*^ and *dy*^*3K*^*/dy*^*3K*^ expressing αLNNd, *mag*, or both (Fig. 5, Suppl. Fig. S7). WT and *dy*^*3K*^*/dy*^*3K*^ phosphorylation of both were similarly low. Both phosphor-mTOR and Akt were elevated in *mag*- *dy*^*3K*^*/dy*^*3K*^ and DT-*dy*^*3K*^*/dy*^*3K*^ muscle, but not in αLNNd-*dy*^*3K*^*/dy*^*3K*^ muscle.

### Laminin subunit detection in muscle extracts:

**Fig. 6.**
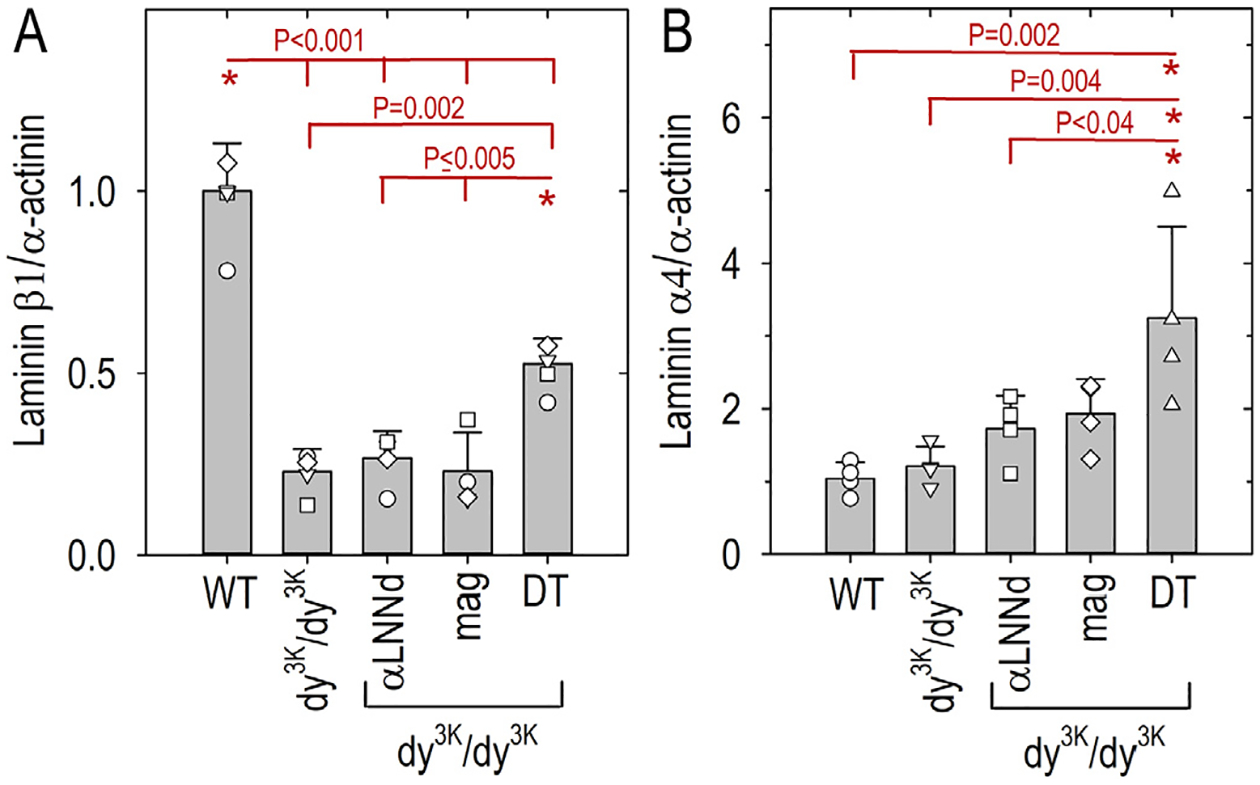
Laminin subunit protein expression elevations in DT-dy^3K^/dy^3K^ muscle extract immunoblots. Forelimb triceps muscle was excised from 3-week-old WT, *dy*^*3K*^*/dy*^*3K*^ and *dy*^*3K*^*/dy*^*3K*^-transgene mice (4 animals per condition), ground, washed with Triton X-100 buffer to remove non-BM laminins, then extracted with SDS/EDTA buffer. The extracts were electrophoresed by SDS-PAGE, trans-blotted, and probed with either rat mAb anti-Lmβ1 (panel A) or chick polyclonal anti-Lmα4 (panel B) antibodies and anti-α-actinin antibody as a loading control (immunoblots shown in supplement). Graphs show the laminin subunit/α-actinin ratios (av. ± s.d.). Individual mouse values (open symbols) are superimposed on the average bars. P values determined by 1-way ANOVA followed by pairwise Holm-Sidak test. DT-*dy*^*3K*^*/dy*^*3K*^ Lmβ1 values were significantly higher than *dy*^*3K*^*/dy*^*3K*^ without or with single transgenes but lower than WT values and Lmα4 values were higher in DT-*dy*^*3K*^*/dy*^*3K*^ compared to WT and mag- *dy*^*3K*^*/dy*^*3K*^.

Hindlimb muscle obtained from three week-old-mice at an age that largely predates the onset of fibrosis was chosen for immuno-biochemical analysis (Fig. 5). To compare the muscle extracellular matrix (“myomatrix” [32]) laminin β1 and α4 subunits among WT, *dy*^*3K*^*/dy*^*3K*^, αLNNd-*dy*^*3K*^*/dy*^*3K*^*, mag-dy*^*3K*^*/dy*^*3K*^, and DT-*dy*^*3K*^*/dy*^*3K*^ 3 week-old mice, excised frozen forelimb muscle ground and washed with Triton X-100 to remove loosely-bound and intracellular proteins. The remaining material was extracted with EDTA/SDS and subjected to SDS-PAGE under reducing conditions and immunoblotted with subunit detection with antibodies specific for the laminin β1 and α4 subunits (Fig. 6 and Supplemental. Fig. S8). The immunoblots revealed no significant increase in either β1 or α4 subunits in *dy*^*3K*^*/dy*^*3K*^ single transgene muscle compared to *dy*^*3K*^*/dy*^*3K*^ in the absence of transgene. In contrast, double transgene (DT) expression in *dy*^*3K*^*/dy*^*3K*^ significantly increased BM laminins by elevating β1 and α4 laminin levels in the myomatrix fraction to levels significantly higher than *dy*^*3K*^*/dy*^*3K*^ muscle without or with single transgenes.

**Fig. 7.**
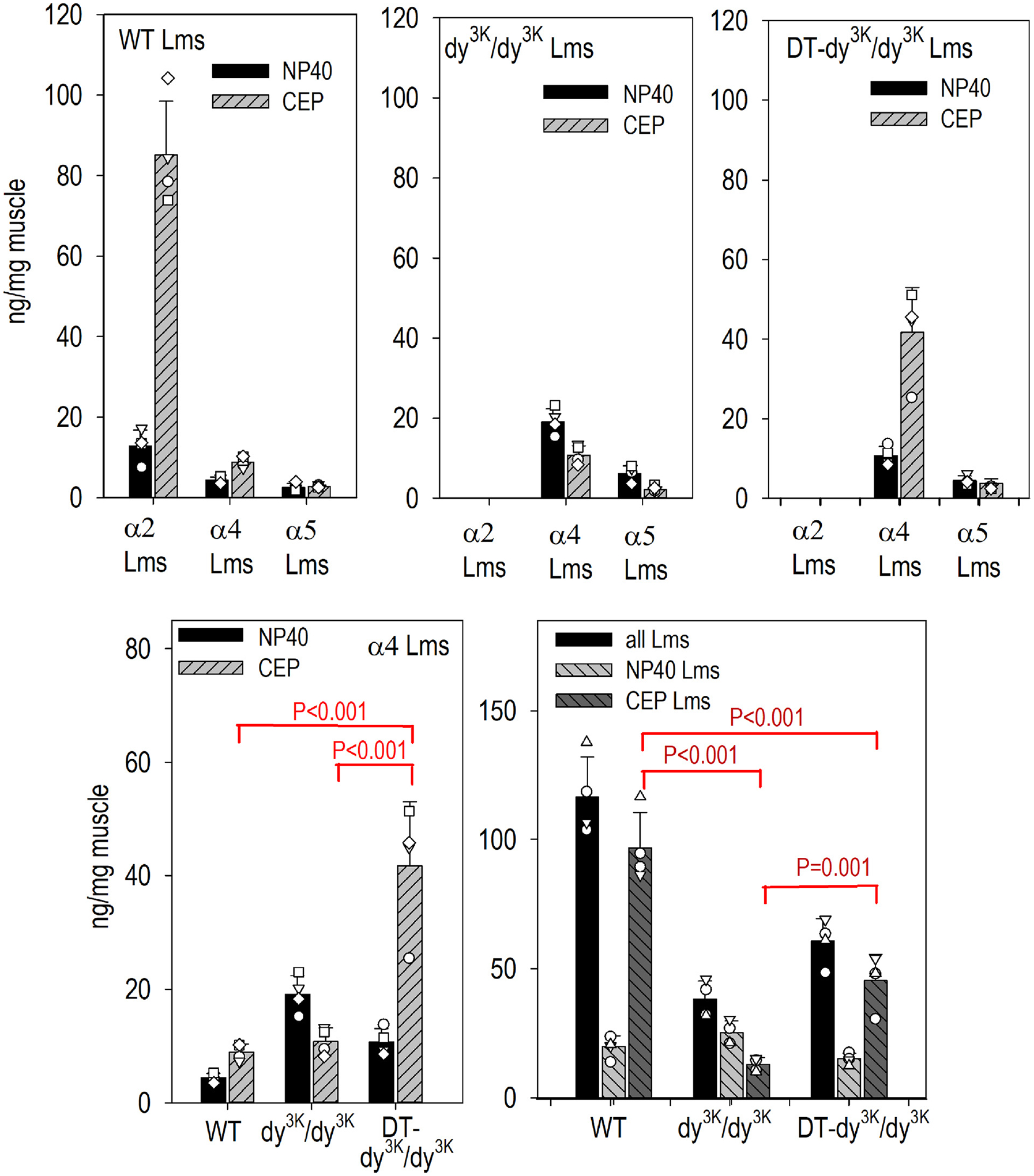
Sandwich ELISA assays of fractional extracts of heterotrimeric muscle laminins. Forelimb muscle from four WT, four *dy*^*3K*^*/dy*^*3K*^, and four DT- *dy*^*3K*^*/dy*^*3K*^ mice at three weeks of age were collected followed by extraction and separation of detergent-soluble (TBS-buffer with NP40) and extracellular matrix laminins (consisting of the combined collagenase-released, EDTA-solubilized, and SDS fractions (designated “CEP”). ELISA sandwich assays, in which the laminins were attached to immobilized antibody to one subunit, followed by detection of laminin with a second antibody to a different subunit, were used to estimate the distribution and amounts of heterotrimeric laminins in the different fractions compared to laminin standards of known concentration as described in the Methods. Av. ± s.d. with individual mouse values (open symbols) shown. α2 laminins (Lm211, Lm221) detected only in WT muscle, were mostly present in the CEP fraction with very small amounts of α4 and α5 laminins. *Dy*^*2J*^*/dy*^*2J*^ muscle contained mostly α4 laminins, slightly increased relative to WT, with the majority present in the detergent-solubilized fraction. DT - *dy*^*3K*^*/dy*^*3K*^ muscle exhibited an increase of α4 laminins in the CEP fraction. Total measured DT- *dy*^*3K*^*/dy*^*3K*^ laminins were elevated to levels approximately midway between WT and *dy*^*3K*^*/dy*^*3K*^.

In a comparison of laminin heterotrimers, muscle was extracted with a sequence of buffers based on an approach developed previously to separate soluble peripheral laminin from myomatrix laminin integral to BM [18]. An initial NP40 neutral salt buffer was used to remove loosely adherent and intracellular laminins. This was followed by sequential a combined collagenase-treatment (“C”) that releases some laminins, EDTA-extraction (“E”) to depolymerize laminins and break receptor interactions, and finally SDS-extraction of the remaining pellet (“P”) to remove more strongly-bound residual laminins. These “CEP” fractions were combined and analyzed by an ELISA-sandwich technique to detect laminin trimers with antibodies to β and α subunits (Fig. 7 and Supplemental Fig. S9). The analysis revealed that (a) myomatrix CEP laminins were the principal fraction in WT and DT- *dy*^*3K*^*/dy*^*3K*^, whereas the NP40 fraction predominated in *dy*^*3K*^*/dy*^*3K*^ without transgenes, (b) WT muscle contained relatively high levels of α2-laminins that were, as expected, not present in *dy*^*3K*^*/dy*^*3K*^ muscle. (c) α4 laminins represented the principal laminins in *dy*^*3K*^*/dy*^*3K*^ muscle with only a small contribution of α5 laminins. In the absence of transgenes, *dy*^*3K*^*/dy*^*3K*^ laminins were reduced to ~1/3 compared to WT laminins (by mass) and α4-laminins were ~30% by mass (~1/3 on molar basis) of WT α2 laminins. (d) Double transgene expression increased the total α4 laminins from ~30% to ~52% by mass (~65% on a molar basis). (e) This increase was in the CEP fraction with a decrease in NP40 fraction. (f) Overall myomatrix laminins reached ~50% of WT laminins levels.

### Linker proteins and laminin subunits visualized in muscle sarcolemma and capillaries.

While embryonic skeletal muscle BMs contain α1, α2, α4 and α5 laminins [33], mature WT muscle sarcolemmal BMs lose all but the α2 laminins with α4 and α5 laminins limited in distribution to intramuscular blood vessel BMs [33,34]. In contrast, laminin-deficient and laminin-null muscle sarcolemmal BMs contain primarily α4 laminins [34] in which α4 and α5 subunits appear to be redistributed from a localized microvascular to a sarcolemma plus microvascular distribution.

**Fig. 8.**
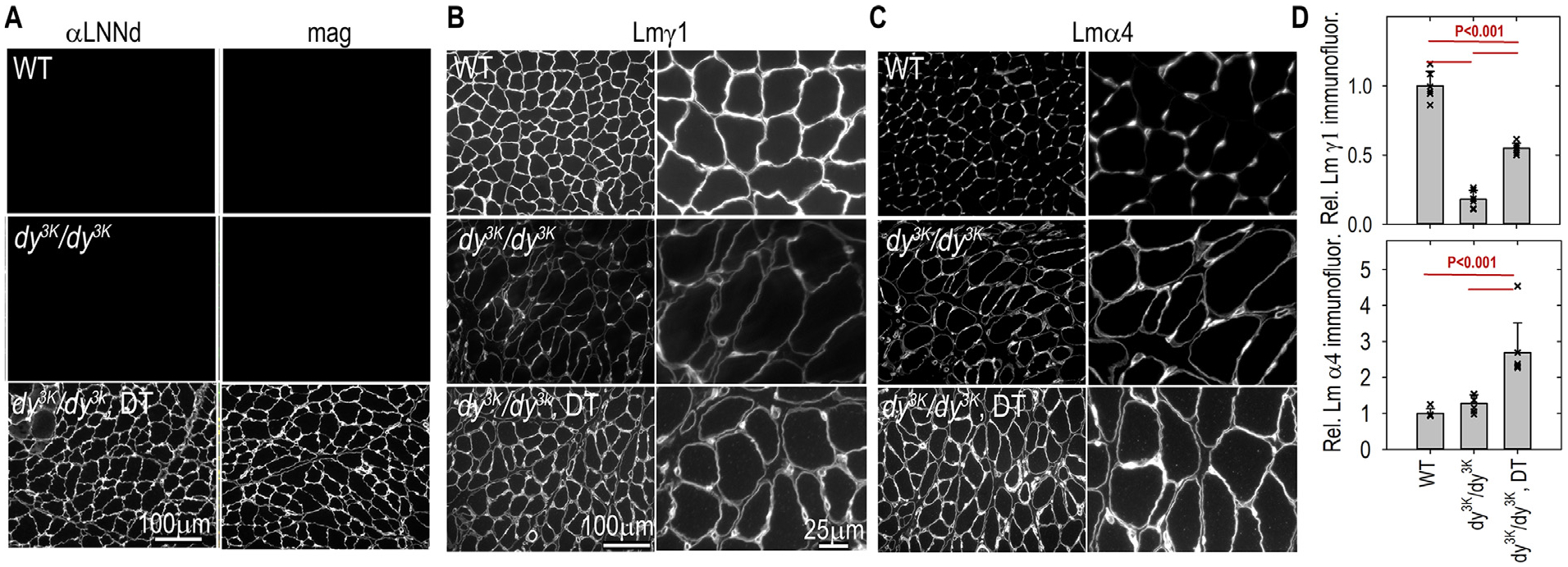
Laminin muscle immunofluorescence. Frozen sections of hindlimb muscle from 3-week-old mice were immunostained with antibodies to detect linker proteins and laminin subunits. Panel A: antibodies to Lmα1 LN-LEa domains (αLNNd) and mag. Panel B: laminin-γ1. Panel C: laminin α4. Panel D: Morphometry comparing the sum of BM intensities (av. ± s.d. with individual image values (symbols) shown. Both subunits were reduced in *dy*^*3K*^*/dy*^*3K*^ muscle. DT-*dy*^*3K*^*/dy*^*3K*^ mice revealed an increase in Lmγ1 to levels about half-way between WT and dy^3K^/dy^3K^ without transgenes whereas Lmα4 levels were significantly higher in DT-*dy*^*3K*^*/dy*^*3K*^ compared to WT and *dy*^*3K*^*/dy*^*3K*^ mice.

Frozen sections of hindlimb muscle from 3-week-old mice immunostained for laminin γ1 (common to all BMs) and laminin α4 were examined (Fig. 8). The BM expression of Lmγ1 in untreated dystrophic muscle were notably reduced compared to WT muscle, evidence for a general decrease in total laminins in dystrophic muscle. In contrast, the Lmγ1 subunit was increased in dystrophic muscle expressing both (DT) transgenes. Laminin α4 expression was slightly increased in untreated dystrophic muscle accompanied by a prominent change in distribution from capillaries to sarcolemma. The sarcolemmal α4 staining was considerably increased in dystrophic muscle expressing both transgenes.

### Muscle immunostaining of other BM zone components and receptors.

Frozen sections of proximal hindlimb muscle from 3-week-old mice were examined by immunostaining for laminin subunits α5, α2, β1 and β2, perlecan, collagen-IV, agrin (Supplemental Fig. S11) and for β1- and α7 integrins and αDG (Supplemental Fig. S12). Lmα5 was detected in the microvasculature in WT muscle that extended to include sarcolemma in *dy*^*3K*^*/dy*^*3K*^ and DT-*dy*^*3K*^*/dy*^*3K*^. However, the ELISA sandwich assays (Fig. 7) led us to conclude that Lmα5 is a minor species relative to Lmα4 in the dystrophy. Lmα2, as expected, was present only in the sarcolemma of WT muscle. Sarcolemmal Lmβ1 (like Lmγ1, Fig. 8) and collagen-IV were decreased in *dy*^*3K*^*/dy*^*3K*^ and increased in DT-*dy*^*3K*^*/dy*^*3K*^. Lmβ2 was mainly detected in the perineurium of sciatic nerve branches (and some vasculature) and was a minor component of muscle sarcolemma while perlecan was present in the different mice with small differences. Endogenous agrin (the antibody does not react with *mag*) was increased in sarcolemmal and microvascular *dy*^*3K*^*/dy*^*3K*^ and DT- *dy*^*3K*^*/dy*^*3K*^. Sarcolemmal β1- and α7-integrins were reduced in *dy*^*3K*^*/dy*^*3K*^ and increased in DT- *dy*^*3K*^*/dy*^*3K*^, suggesting that the BM laminin reduction and increments causes parallel changes in α7β1 integrin. Sarcolemmal αDG was moderately increased in both *dy*^*3K*^*/dy*^*3K*^ and DT- *dy*^*3K*^*/dy*^*3K*^ relative to WT.

### Histology of muscle from a one-year-old surviving DT-dy^3K^/dy^3K^ mouse.

Fixed and paraffin-embedded forelimb and hindlimb muscles from the mouse were stained with PAS and PSR. Plantaris, TA and triceps images are shown (Supplemental Fig. S13). The extent of fibrosis was similar to that observed at six weeks. The fraction of central nuclei were even somewhat higher, suggesting that regeneration continued for months without depletion of the pool of satellite cells.

## Discussion

The αLNNd and *mag* muscle-specific transgenes each exerted a limited amelioration of the muscular dystrophy of the *dy*^*3K*^*/dy*^*3K*^ mouse seen mostly as a doubling of survival. When the two transgenes were combined, survival time was tripled, weights were increased, and improvements in muscle histology that included increases in myofiber number, whole muscle cross-sectional area, average individual myofiber size, and reduction of fibrosis were noted. However, the elevated dystrophic regeneration (as reflected in the fraction of central nuclei) was not reduced, suggesting that continued regeneration was required to maintain the increase in muscle mass. Evidence for continued regeneration with age and retained regenerative capacity is suggested by the high central nucleation that was observed in a one-year-old DT-*dy*^*3K*^*/dy*^*3K*^ mouse. Finally, while the measured increases in forelimb and all-limb grip strength were significant, they were still well below that of wild-type mice.

The observed amelioration correlated with increases in muscle BM laminin. Analysis of total γ1 or β1 laminin in muscle by immunofluorescence intensity and immunochemical analysis of muscle laminins revealed that the dystrophic muscle contained only about one-quarter of the laminin in WT muscles in the myomatrix fraction representing BM and adjacent stroma. This increase was seen mostly in the subunits of Lm411. Further, WT muscle compared to dystrophic muscle contained a larger fraction of laminins in the myomatrix BM compared to the detergent-wash fraction, evidence that the predominant Lm411 species is poorly retained in BM. This laminin isoform property can be explained by previous analyses that revealed that α4-laminins neither polymerizes nor binds to αDG, rendering it poorly adherent to cell surfaces, self, and other BM components [8]. Further, Lm411 binds weakly to α7β1 integrin compared to Lm211 [8,35]. The expectation in providing the two transgenes to the dystrophic mice was that enabling polymerization with αLNNd and enabling binding to the αDG receptor would stabilize the laminin layer through the formation of lateral linkages that enhance cooperativity of association and that increase anchorage to the myofiber surface. This combination of modifications would increase laminin incorporation into the BM (“capture”) and decrease the laminin lost through dissociation of weak bonds from the myofiber surface and BM. The finding of a substantial increases in α4-laminins in BMs with an accompanying decrease in the detergent fraction through dual-linker transgenesis supports this expectation. While it is possible there is an additional contribution through increased transcription and/or synthesis (Lmα4-transcription was only found to trend higher in the DT-*dy*^*3K*^*/dy*^*3K*^), that would not explain the shift from the detergent to the matrix fraction.

The normally high levels of laminin expression in BMs demands a high level of linker protein expression since the assembly process is stoichiometric rather than catalytic. Therefore, the level of transgenic expression of linker proteins relative to that of laminins likely greatly determine the amount of functional BM laminin. The creatine kinase promoter, while effective, is not an optimal one for high muscle expression. For example, in a comparison of muscle-specific vs. universal (CBh) expression by AAV-mediated transgenesis of the *dy*^*2J*^*/dy*^*2J*^ mouse dystrophy, considerably higher expression and phenotypic benefit was achieved with the latter [36].

The increase in muscle fiber diameter and in mTOR and Akt phosphorylation observed in the *dy*^*3K*^*/dy*^*3K*^ mice with the *mag* transgene alone, but not with the αLNNd transgene alone, suggests that those changes are mediated through α4-laminin binding to αDG rather than through its polymerization. If only polymerization occurs, αLNNd-α4-laminin is still unable to bind αDG and bind only weakly to α7β1 integrin to form linkages to the cytoskeleton and transduce signals. Phosphorylated mTOR and Akt values may be higher in *mag*-treated muscle compared to WT because there is an ongoing process of active myofiber death and regeneration in the former but not the latter state. In another study, disrupting the interaction between αDG and laminin was found to induce apoptosis and reduce Akt phosphorylation [37] while treatment of a fukutin-deficient dystroglycanopathy with the mTOR inhibitor RAPA was found to increase myofiber size [38]. Thus, a similar misregulation of mTOR and Akt signaling appears to exist in both fukutin- and Lama2-deficiency, one that might be correctable by restoration of laminin binding to αDG.

Overall, co-expression of the two transgenes produced an amelioration of the severe disease that was substantial, but not as robust as that achieved with the same transgenes and promoter in the less severely-affected hypomorphic *dy*^*W*^*/dy*^*W*^ mouse [8]. Further, the amelioration was even less than that achieved by transgenic expression of the entire Lmα1 subunit driven by a strong universal (CAG) promoter [25]. The *dy*^*W*^*/dy*^*W*^ mouse is not a full knockout in that it expresses small amounts of a truncated laminin that lacks the LN (domain “VI” by the old nomenclature) domain [20]. Muscle-specific linker protein repair, previously evaluated in *dy*^*2J*^*/dy*^*2J*^ and *dy*^*W*^*/dy*^*W*^ mice, has now been evaluated in *dy*^*3K*^*/dy*^*3K*^ mice, revealing a spectrum of striking to modest responses correlating with disease severity. The difference in Lmα2 expression among the mouse models may be the most important factor accounting for the degree of disease severity and response to linker expression. Half of untreated *dy*^*3K*^*/dy*^*3K*^ died by 4 weeks while half of the *dy*^*W*^*/dy*^*W*^ mice died by about 18 weeks in a linker protein study [8]. *Dy*^*2J*^*/dy*^*2J*^ mice, on the other hand, exhibit only a slight reduction in laminin expression and are long-lived. Treatment of these mice with a single linker protein, αLNNd, ameliorated the muscle phenotype such that forelimb muscle strength was similar to that of normal mice [18]. The amelioration response in *dy*^*2J*^*/dy*^*2J*^ and *dy*^*W*^*/dy*^*W*^ mice to double transgene treatment (αLNNd and *mag*) was less than single transgene expression in *dy*^*2J*^*/dy*^*2J*^. Further, amelioration was not identical in the two severely affected mouse models in that survival was extended three-fold in *dy*^*3K*^*/dy*^*3K*^ and five-fold in *dy*^*W*^*/dy*^*W*^. On the other hand, the increases in weights were similar in both with DT with dystrophic mice reaching about 15 grams by 10 weeks of age compared to about 22 grams for WT mice. Although measured by different types of assays, total laminin levels appeared to be considerably less in the *dy*^*3K*^*/dy*^*3K*^ compared to the *dy*^*W*^*/dy*^*W*^ mice (nearly the same as WT). Of note, the surviving *dy*^*W*^*/dy*^*W*^ Lmα2 subunit shares a structural feature with *dy*^*2J*^*/dy*^*2J*^ Lmα2 in that both lack the α2LN polymerization domain. They differ, of course, in that the *dy*^*W*^*/dy*^*W*^ mice express very little α2 laminin whereas the *dy*^*2J*^*/dy*^*2J*^ mice express only slightly reduced levels of α2 laminin. However, since αLNNd repairs polymerization by providing a missing LN domain, one would predict that it would improve the phenotype in *dy*^*3K*^*/dy*^*3K*^ only by enabling α4 laminin polymerization whereas it would improve the phenotype in *dy*^*W*^*/dy*^*W*^ by enabling polymerization of the residual α2 laminin as well as by α4 laminin. To what extent this difference accounts for differences in disease amelioration is unclear.

The approach to therapy suggested by the muscle-specific current study uses small proteins that add needed functional activities to compensating α4 laminins. The DNAs coding for these proteins are able to fit, along with a high-expression promoter and poly-A tail, within the small capsid of adeno-associated virus [36,39]. A superior repair for Lama2- dystrophy was achieved in *dy*^*3K*^*/dy*^*3K*^ mice by universal transgenic expression of the entire laminin α1 subunit using the high-expression CAG promoter [25,30,40,41]. The Lmα1 subunit has a domain structure that is identical to the absent Lmα2 such that as trimeric laminin polymerizes and binds to the α7β1 integrin and to αDG. Specifically, the transgenic Lmα1 dystrophic mice exhibited near-normal grip strengths and weights even though by adulthood exhibited high regeneration rates in several muscles. While a limitation of the initial studies is that the transgenic DNA coding for the subunit is far too large (~9 kB DNA) to be delivered to a dystrophic host by other means, a recent study demonstrated that AAV-CRISPR-Cas was used to activate the Lama1 gene in muscle and peripheral nerve in *dy*^*2J*^*/dy*^*2J*^ mice with substantial amelioration of the dystrophic phenotype comparable to that achieved by AAV-CBh-αLNNd [36,42]. Presumably this novel alternative approach can also be used to treat the *dy*^*W*^*/dy*^*W*^ and *dy*^*3K*^*/dy*^*3K*^ mouse models, possibly achieving high WT weights and grip strength. Improvements in survival, weight and strength may depend on two factors. The first is that high expression is needed for a stoichiometric structural repair. A second factor is that addition of high affinity α7β1 binding may further contribute to phenotypic improvement as well. Further optimized studies comparing linker protein to whole laminin subunit repair will be needed to determine the most important factors.

## Methods

Protein determinations: Molar laminin concentrations were determined by densitometry of Coomassie blue-stained acrylamide gels in comparison to an EHS-laminin (710 kDa protein mass) standard ([43]) corrected for changes in calculated mass (LmΔαLN-L4b, 558 kDa), Lm-411 (513 kDa), Lm-511 (784 kDa) and Lm-211 (718 kDa) as previously described ([17,44]). Absorbance at 280 nm was used to measure the concentration of αLNNd (156 kDa) and miniagrin (136 kDa).

### Mice and genotyping:

(a) *Dy*^*3K*^/+ mice, originally in a C57Bl/6 background, were bred with 129SvEv-Tac mice (Taconic Labs) to generate hybrid mice with improvements in litter frequency and size. (b) For genotyping at the time of weaning, genomic DNA was purified from small pieces of mouse tail in 0.1 M NaOH buffer and boiled at 100°C in PCR tubes in the thermocycler for 10 min. Samples were cooled, vortexed and immediately diluted into 40 mM Tris buffer, pH 8.0. Genotyping PCRs were done with one ml of genomic DNA per 20 ml reaction according to the Jumpstart Taq (Sigma P2893) instructions.

### Microscopy and Morphometry:

Forelimbs and hindlimbs were dissected from euthanized mice and processed basically as described [36]. (a) For paraffin embedding, muscles were fixed in 10% buffered formalin overnight (Sigma SF93–4). Specimens were then incubated overnight at RT with an acidic decalcification solution (Fisher Cal-Ex CS510–1D), washed in running tap water for several hours, re-fixed in 10% formalin overnight, rinsed, cross-sectioned, and maintained in 70% ethanol for tissue processing. Tissues were embedded, sectioned at 5 μm, and stained with periodic acid Schiff (PAS) and Picro-Sirius Red (PSR) at the Histopathology Core Pathology Services Rutgers University). Panoramic images were recorded with a Leica Slide Scanner and analyzed with Aperio ImageScope software. (b) For frozen sections, unfixed muscles were embedded in OCT (Tissue-Tek, Elkhart, IN) and flash frozen in liquid nitrogen. Five-micron thick sections were cut with a cryostat (Leica CM 1850) at −20 °C and adhered to positively charged slides (Fisher). Sections were then washed for 5 min in TBS-50 followed by fixation in 3.2 % paraformalde-hyde in PBS for 15 min at room temperature. Slides were washed in PBS and blocked in 5% goat serum overnight at 4 °C. Primary antibodies were added the following day for 1–3 h at room temperature and washed 3 times in PBS for 10 min. Secondary antibodies conjugated with fluorescent probes were added for 1 hour at room temperature, followed by 30 min of PBS washes changed every 10 min. Slides were mounted with coverslips in 6% DABCO (1,4-diazabicyclo[2.2.2]octane) in glycerol. Detection of bound primary antibodies in fixed frozen sections was accomplished with Alexa Fluor 488 and 647 goat anti-rabbit, chicken, and mouse IgG secondary antibodies (Molecular Probes) at 1:100. Tissue sections were stained together with antibodies tittered to ensure linear detection of basement membrane components. Regions of muscle were matched between genotypes, and the same exposure times and normalizations were applied to all images being compared. (c) For electron microscopy, skeletal muscle was fixed in 0.5% gluteralde-hyde and 0.2% tannic acid in PBS for 1 h, washed with 0.1 M sodium cacodylate buffer, transferred to modified Karnovsky’s fixative, post-fixed in 1% osmium tetroxide for 1 h, and prepared and imaged as previously described [29].

### Antibodies:

(a) An antibody specific for the laminin-a2 N-terminus (Sigma L0663 rat 1 μg/ml) was used to detect laminin-211 levels in mouse muscle as described [18]. (b) Antibodies to αLNNd were prepared in rabbit against recombinant mouse αLN-Lea [36] and used at a 1 μg/ml dilution for immunostaining. (c) Antibody to miniagrin was prepared in rabbits against recombinant chicken miniagrin (7) and affinity-purified on a chicken miniagrin Sepharose-4B column. (d) An antibody specific for the laminin-a4 subunit was employed at 1 μg/ml, [18]. (e) Rabbit polyclonal laminin-α5 antibody used at 1 mg/ml as described [18]. (f) Monoclonal rat laminin-β1 antibody was used at 0.5 μg/ml (Invitrogen MA5–14657). (g) The laminin-γ1 subunit in mouse tissues was detected by a rat anti-mouse antibody (1914P, Millipore, 1:100). In the myotube laminin assembly assays, a mouse anti-human laminin-γ1 monoclonal was used (Millipore mAb1920, 1:100). (h) Antibodies to evaluate mTOR, Akt and FAK were as follows: Rabbit anti-phospho-Ser2448 mTOR 1:1000 (Cell Signaling 5536s), rabbit anti-mTOR 1:1000 (Cell Signaling 2983s), rabbit anti-Ser473 Akt 1:2000 (Cell Signaling 4060s), rabbit anti-Akt 1:1000 (Cell Signaling 4691s), rabbit anti-phospho-FAK Tyr 925 (Cell Signaling 3284s), rabbit anti-FAK 1:1000 (Cell Signaling 3285s), 1:3000 anti-rabbit HRP (Invitrogen 65–6120), mouse anti α-actinin 1:2000 (Millipore A7811), and 1:3000 anti-mouse HRP (Invitrogen 62–6520). (i) F4/80 monoclonal rat anti-mouse antibody (1:100; Abcam, catalog no. ab6640) was used to detect macrophages in muscle frozen sections. Sections were counterstained with rabbit anti perlecan antibody (1 μg/ml; [45]). (j) Frozen sections of muscle were incubated with the following: Rabbit anti-perlecan (1 μg/ml or 1:100) and rabbit anti-collagen IV (0.5 mg/ml, Millipore AB756p) with detection with 1:100 anti-rabbit Alexa-488 (Invitrogen A11034); Rat anti-β1 integrin antibody (10 μg/ml, Millipore MAB1997) with detection with 1:100 anti-rat Alexa-647 (Invitrogen A21247); Rat anti-α7 integrin antibody (5 μg/ml, R&D Systems 334908) with detection with 1:100 anti-rat (Alexa 647); mouse anti-αDG (1:100 Upstate 05–298) or mouse anti-agrin (5 μg/ml Millipore MAB502) each detected with 1:100 anti-mouse Alexa-647 (Invitrogen A21236). The mouse agrin antibody did not react with chick *mag* in an ELISA assay (0.02 to 10 μg/ml antibody; 10 μg/ml protein coat) in contrast to rabbit- anti chick *mag* which did react with chick *mag* (reaching half-maximal binding at 0.1 μg/ml). Laminin a5 was stained with 1 mg/ml chicken anti-Lmα5 and detected with 1:100 anti-chicken Alexa-647 (Invitrogen A32933); rabbit antii-Lmβ2 (1:1000, gift from Jeff Miner [46]) and detected with 1:100 anti-rabbit Alexa-488.

### Extraction and fractionation of laminins from muscle:

*Method A*: This method of extraction was used for the ELISA sandwich assay to detect heterotrimeric laminins and is based on that as described [18]. Briefly, mouse muscle fragments of equal mass were homogenized (Polytron) in 2 ml of ice cold TBS-50 (50 mM Tris, 90 mM NaCl, 1% NP40, pH 7.4 with protease inhibitors (Sigma P8340)). Homogenates were transferred to 2 ml Eppendorf tubes and centrifuged (13,000 rpm, 10 min, 4°C). The supernatants (NP40 buffer fraction) were saved and the pellets, after washing with fresh TBS-50 buffer, were suspended in a collagenase solution (0.2 mg/ml bacterial collagenase (Worthington CLSPA) in TBS-50, 2 mM calcium chloride, protease inhibitors), incubated at 37 °C for 1 h with frequent agitation, and centrifuged (20 min) with the supernatant saved (collagenase fraction). Pellets were then suspended in ice-cold EDTA buffer (20 mM EDTA in TBS-50, protease inhibitors), rocked overnight at 4 °C, centrifuged (20 min, 4 °C) and supernatants saved (EDTA fraction). The final pellet residue was then extracted with Laemmli SDS solubilizing buffer and the supernatant (SDS fraction) saved after centrifugation. The collagenase (“C”), EDTA (“E”) and SDS pellet (“P”) fractions were combined (“CEP”) and used for determination of matrix-bound laminins. *Method B*: This method was used for immunoblot detection and comparison of single laminin subunits. The initial steps through the NP40 extraction step are as described above using Triton x-100 and the addition of phosphatase inhibitors (Sigma P0044). The remaining pellet was then extracted with buffered SDS/EDTA (2 ml of ice-cold TBS-150 (50 mM Tris, 150 mM NaCl, 1 mm EDTA pH 7.4) 1%SDS, 1% Triton x-100, with protease inhibitors (Sigma P8340)) to release matrix-bound laminins.

### ELISA sandwich assay of heterotrimeric laminins in muscle extracts:

Ninety-six well high protein-binding Costar plates were coated (1–2 μg/ml) with rabbit anti-β1/γ1 laminin (2 μg/ml). The wells were then washed and blocked with blocking buffer (PBS, 1% BSA, 0.06% Triton X100). Two-fold serial dilutions of muscle extracted protein NP40 and CEP fractions were prepared in PBS containing 1% BSA and 1% NP40 and applied (0.1 ml; 100% lysate = 1.25 mg extract protein) to the laminin antibody-coated wells. After incubation for 2 hrs (RT), unbound protein extract was removed by washing. An α-subunit-specific antibody was then added to each well for an hour. The wells were again washed, followed by addition anti-chicken-IgY or anti-goat IgG coupled to HRP (1:5000) to detect bound α-subunit specific antibody. Plates were washed again, and then incubated with TMB color reagent for 3 min. Absorbance was measured at A450 following addition of sulfuric acid to halt further color development. The use of serial dilutions for unknowns rather that same-dilution replicates allowed for determination of whether the unknowns fell into the near-linear initial range of the assay. A standard recombinant laminin of known concentration was separately added to two rows of antibody-coated wells for each plate. Bound laminin was detected by the same antibody reagent used to detect the unknown wells. An apparent dissociation constant (Kd) and Bmax value was determined from replicates of the laminin standard and used to covert absorbance values to ng/well values (ng=(Kd*A450)/(Bmax-A450)). Four lysate values (6.25, 12.5, 25, 50 %) from the fitted sample plot were converted to mass of protein (ng) with the above equation and averaged. For determination of α2-laminins the ELISA plate wells were coated with polyclonal rabbit anti-laminin β1,2/γ1 antibody (4 μg/ml) with lysate detected with monoclonal anti-laminin N-terminal antibody. Recombinant Lm211 was used as standard. For estimation of α4-laminins, plate wells were coated with rabbit laminin β1,2/γ1 antibody (4 μg/ml) with lysate detected with chicken anti-laminin α4 antibody (1 μg/ml). Recombinant Lm411 was used as standard. For estimation of α5-laminins, plate wells were coated with polyclonal rabbit anti-laminin β1,2/γ1 antibody with lysate detected with chicken anti-Lmα5 antibody (1 μg/ml). Recombinant Lm511 was used as standard.

### Statistics:

Averages (Av) and standard deviations (s.d.) were calculated from measured values obtained from three or more images and morphometric measurements of perimeters and areas with the statistical package in SigmaPlot 12.5 or Excel. Averages and standard errors of the mean (s.e.m.) were determined from the means of consecutive sets of determinations (e.g. grip-strength) from different mice and from the means of myofiber cross-section areas from different mice. Three or more conditions were compared by one-way ANOVA followed by Holm-Sidak pairwise analysis in Sigma-Plot. A difference was considered significant for P values ≤ 0.05 and trending towards significance for P values >0.05 – ≤0.10.

### Study Approvals:

The mouse protocol (9999–00384) for the study was approved by the Rutgers University – Robert Wood Johnson Medical School IACUC Committee, Piscataway, NJ. The biosafety protocol (IBC# 13–574) for the study was approved by the Institutional Biosafety Committee of Rutgers University, Piscataway, NJ.

## Supplementary Material

1

2

3

## Data Availability

Data will be made available on request.
