# Supplementary material for: Dual transgene amelioration of Lama2-null muscular dystrophy
Source: Matrix Biol. Author manuscript; Available in PMC 2024 Jan 7. (PMC10771811; doi:10.1016/j.matbio.2023.03.001)
Supplement: 1 [file NIHMS1954119-supplement-1.docx]

**Dual transgene amelioration of Lama2-**

**null muscular dystrophy**

**Karen K. McKee^1^ and Peter D. Yurchenco^1^**^†^

(1) Robert Wood Johnson Medical School, Rutgers University, Piscataway, NJ, USA

**SUPPLEMENTAL DATA**


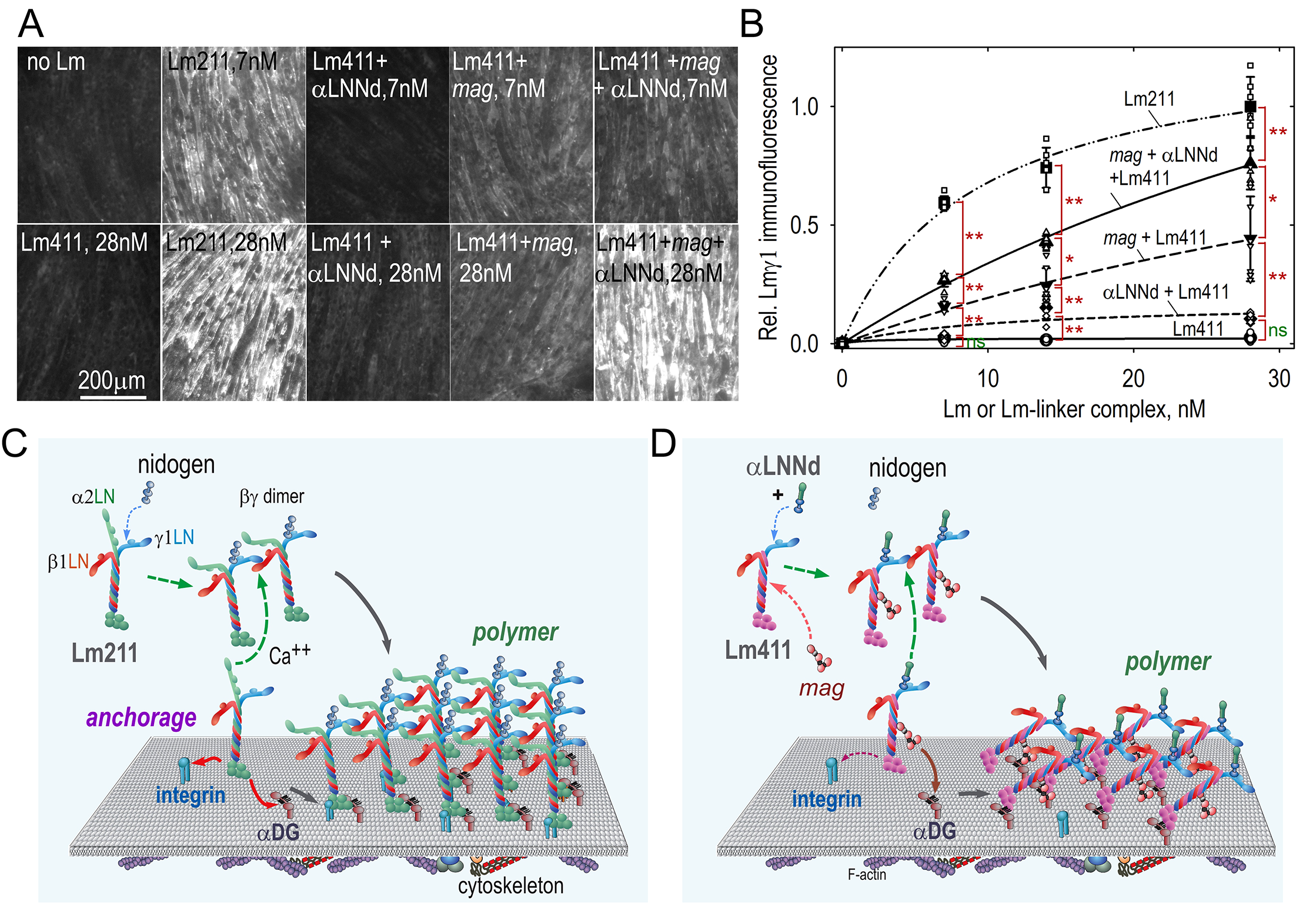


**Supplemental Figure S1.** *Effect of αLNNd and mag linker proteins on laminin assembly on cultured myotubes.* **A, B**. Laminin-211 or laminin-411 without and with the indicated bound linker proteins were incubated in the presence of 28 nM nidogen-1 and 14 nM collagen-IV for 1 hour on cultured C2C12 myotubes. After washing, the cell layers were incubated with monoclonal antibody that binds to the Lmγ1 subunit followed by detected with a fluorescent secondary. Examples of fields shown in A. The sum of fluorescence intensities for each field (n=6-7) was measured and plotted against the incubation concentration. The average, s.d. and individual values are shown. Significance was determined from pairwise comparisons using the Holm-Sidak method (** P<0.001; * P≥0.005; ns, not significant). **C, D**. The data are compatible with the models for Lm211 and Lm411 assembly with both linker proteins. Lm211 polymerizes and binds to integrin α7β1 and αDG while Lm411 is unable to polymerize (lacks an αLN domain), binds poorly to integrin and does not bind to αDG. αLNNd, upon binding to Lm411, enables polymerization. Miniagrin (*mag*), upon binding to Lm411, enables binding to αDG. Together, the linker proteins, attached to Lm411, drive cell surface assembly. The relative lengths of laminin coiled-coil and mag and the location of binding loci suggests the laminin polymer plane would be close to the cell surface.


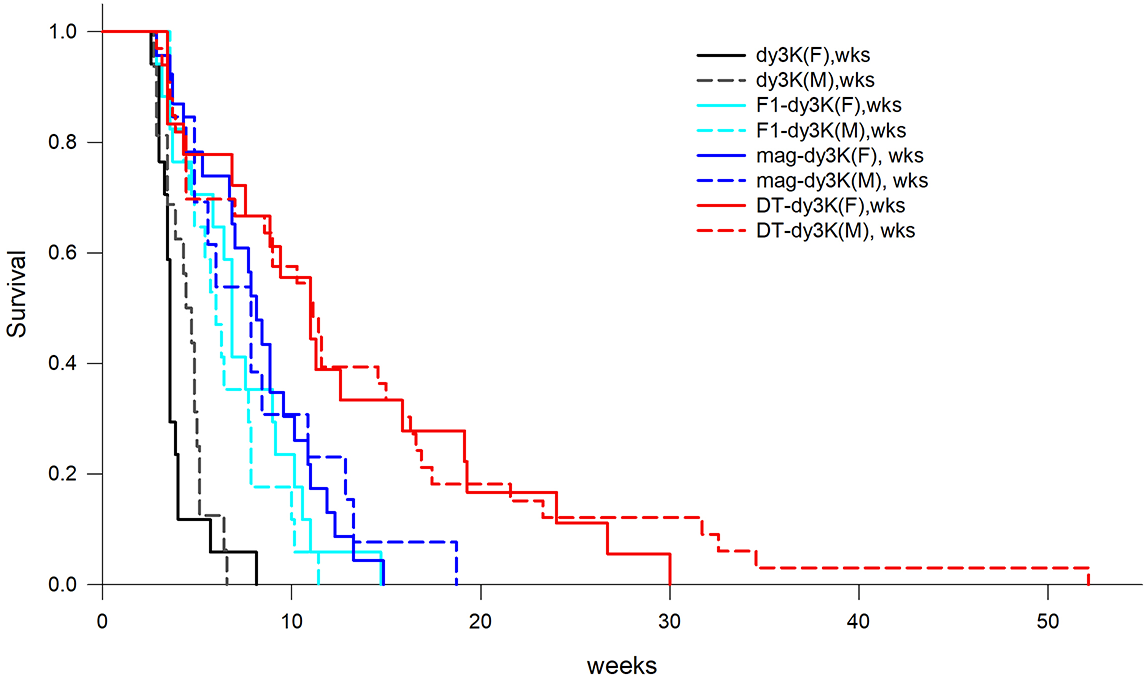


**Supplemental Figure S2.** Kaplan-Meir Plot of Survival by Sex. The survival of *dy^3K^/dy^3K^* males and females is compared in this graph. Significant differences between sexes were not identified for dystrophic mice with double transgene, single transgene and no transgene expression.


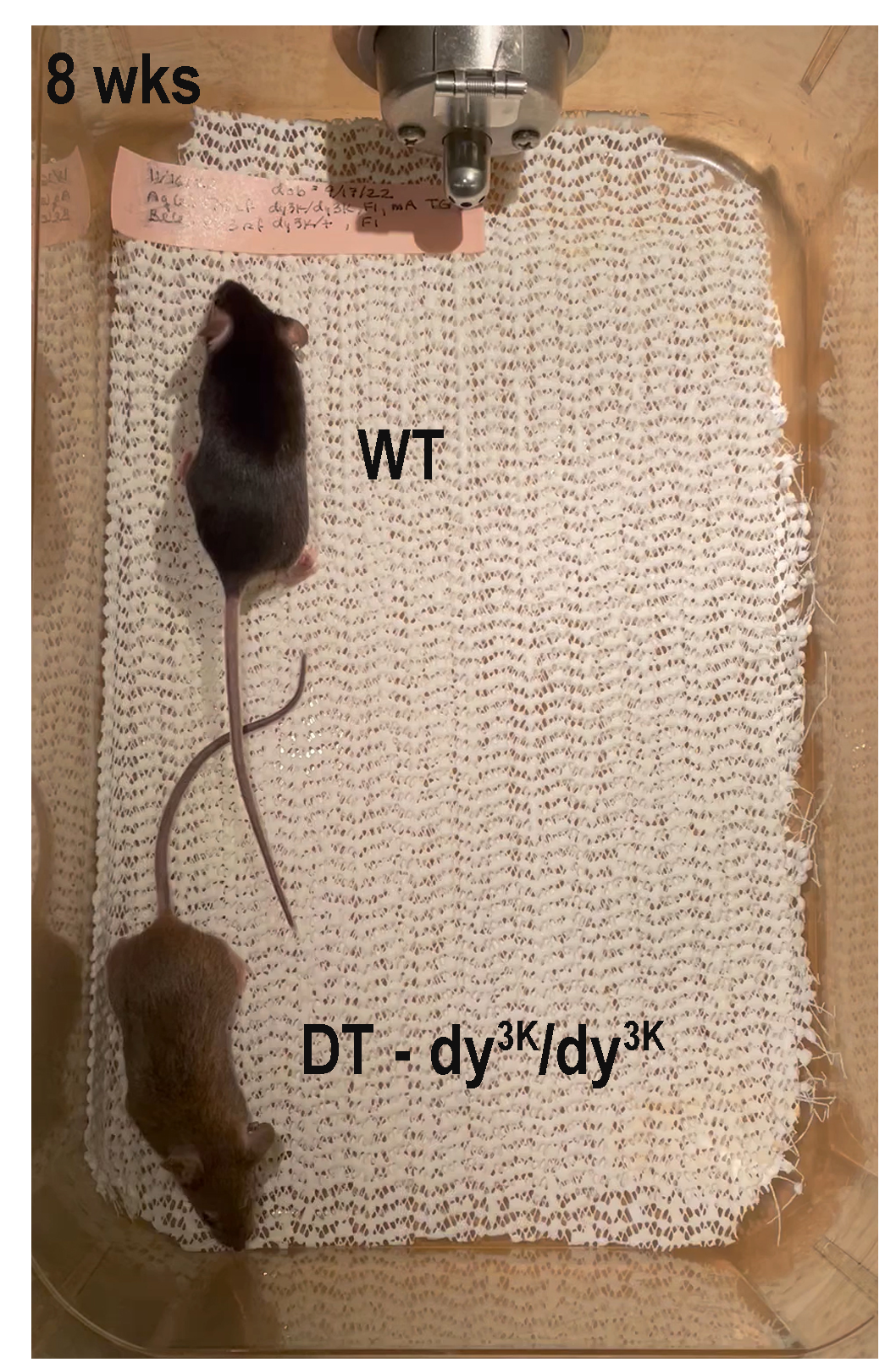

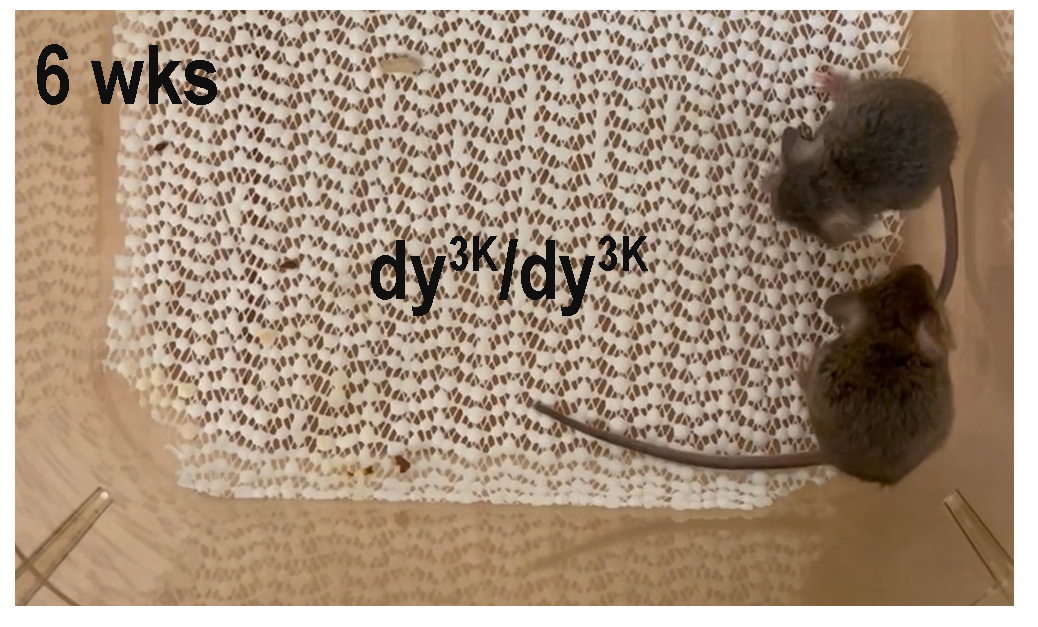


**Supplemental Figure S3.** Representative images of *dy^3K^/dy^3K^* mice at six weeks age (upper image) and WT and DT - *dy^3K^/dy^3K^* mice at eight weeks age (lower image). *Dy^3K^/dy^3K^* mice without transgene show limited mobility and do not survive long. DT-*dy^3K^/dy^3K^* mouse exhibit early hindlimb gait abnormality that leads to later hindlimb extension contractures. Videos are available in Supplement.


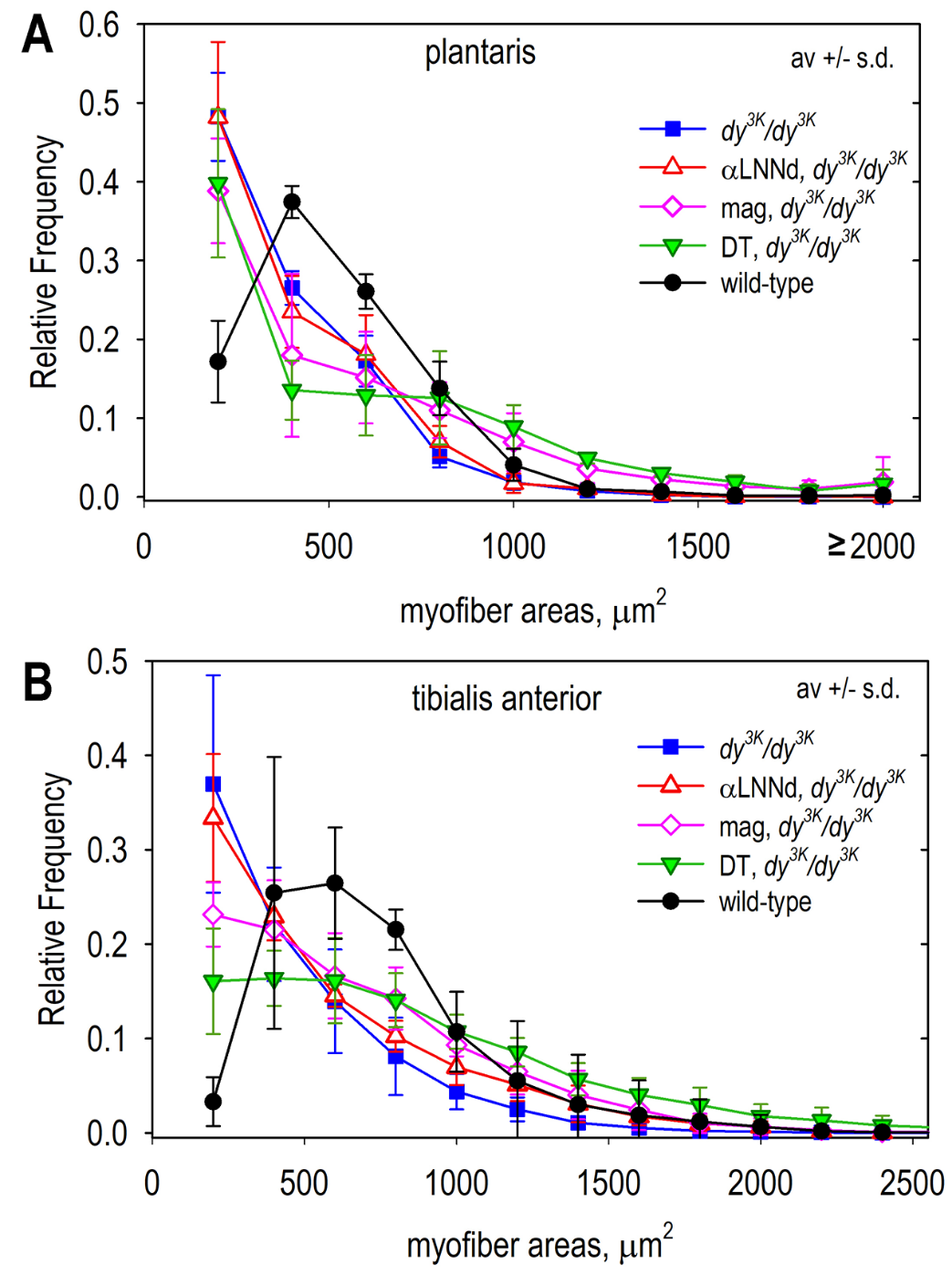


**Supplemental Figure S4.**  *Myofiber area histograms.* Distribution of myofiber cross-section areas (average and s.d.) for plantaris (A) and tibialis anterior (B) from wild-type (n=4, 5 in A and B), *dy^3K^/dy^3K^*, αLNNd (n=5), *dy^3K^/dy^3K^* (n=5, 6 in A and B), mag, *dy^3K^/dy^3K^* (n=5), and DT, *dy^3K^/dy^3K^* (n=5, 6 in A and B) hindlimb muscle of 6-week-old mice. The *dy^3K^/dy^3K^* myofiber distribution contains a large fraction of small myofibers compared to WT. The αLNNd- *dy^3K^/dy^3K^* myofiber distribution is similar or slightly shifted to larger size while the *mag-dy^3K^/dy^3K^* myofibers are larger. This shift to large is greater with the DT*-dy^3K^/dy^3K^* myofibers.


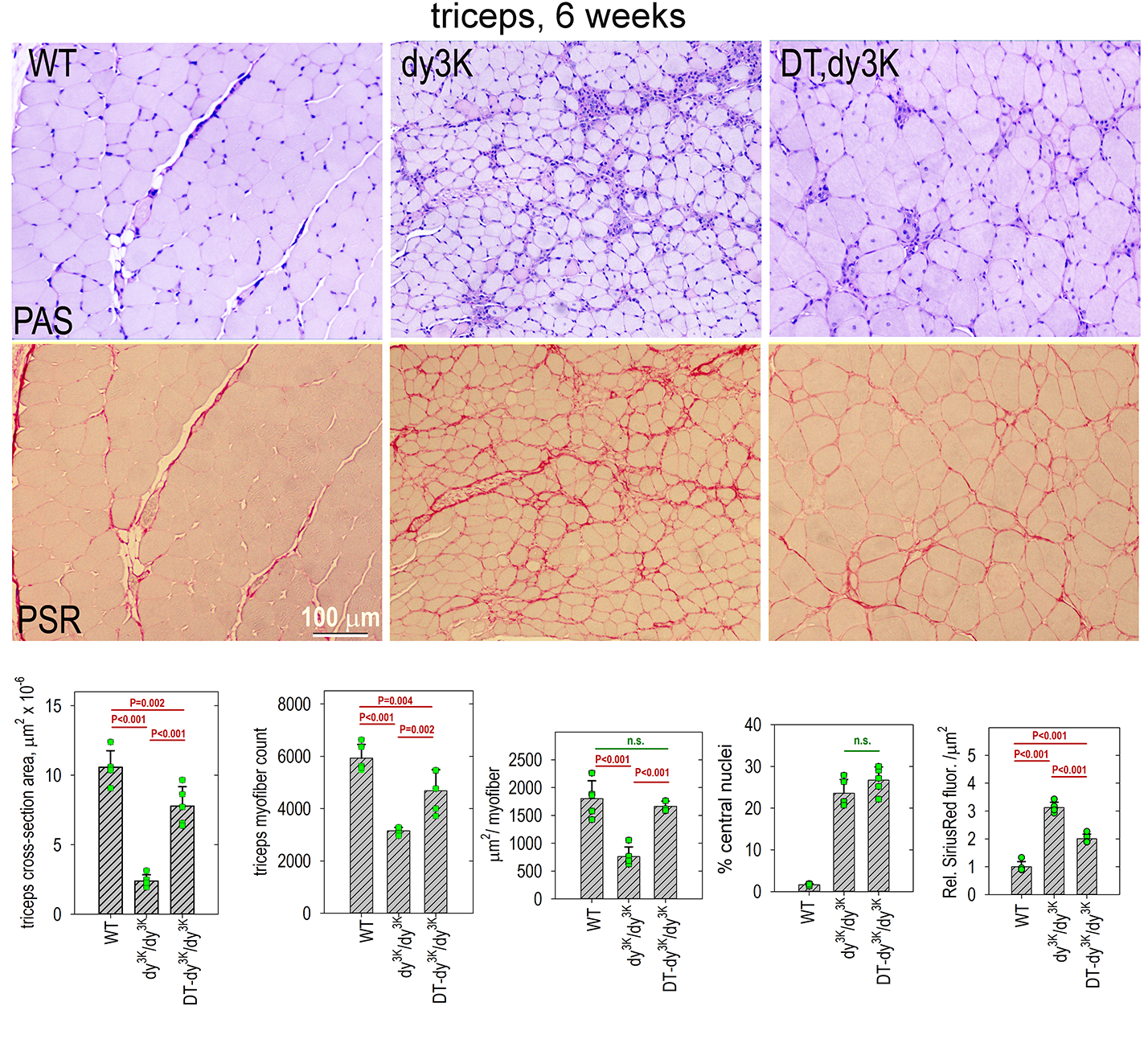


**Supplemental Figure S5**. *Histopathology of WT and dystrophic triceps*. Muscle, harvested from five WT, five *dy^3K^/dy^3K^* (“dy3K”), and five DT- *dy^3K^/dy^3K^* mice at 6 weeks of age, were analyzed after staining with PAS and Picro-Sirius red. Average, s.d. and individual mouse values shown. Significance was determined from pairwise comparisons using the Holm-Sidak method. The DT muscle showed improved number of fibers in triceps, average myofiber cross-sectional areas, and overall muscle area.


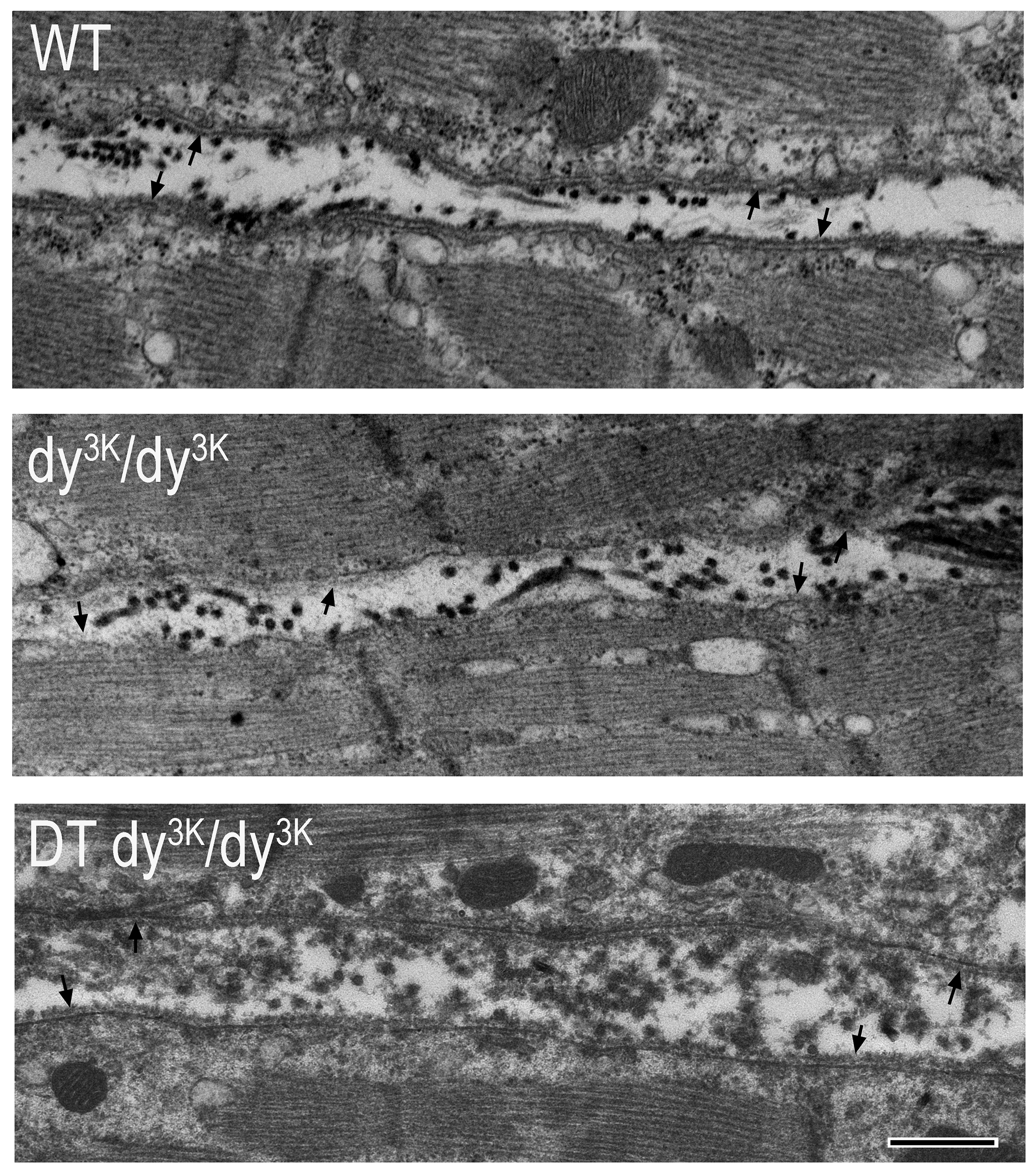


**Supplemental Figure**. **S6.** *Muscle basement membrane ultrastructure.* Hindlimb muscle from 3-week-old mice showing the extracellular matrix of adjacent myofibers. Arrows indicate sarcolemmal BMs and intervening interstitial collagen fibers (mostly in cross-section). Magnification bar, 500 nm. The *dy^3K^/dy^3K^* BMs are rarified (less electron dense) compared to WT. In contrast, the BMs of the DT*-dy^3K^/dy^3K^* muscle exhibits increased electron density approaching that of WT.


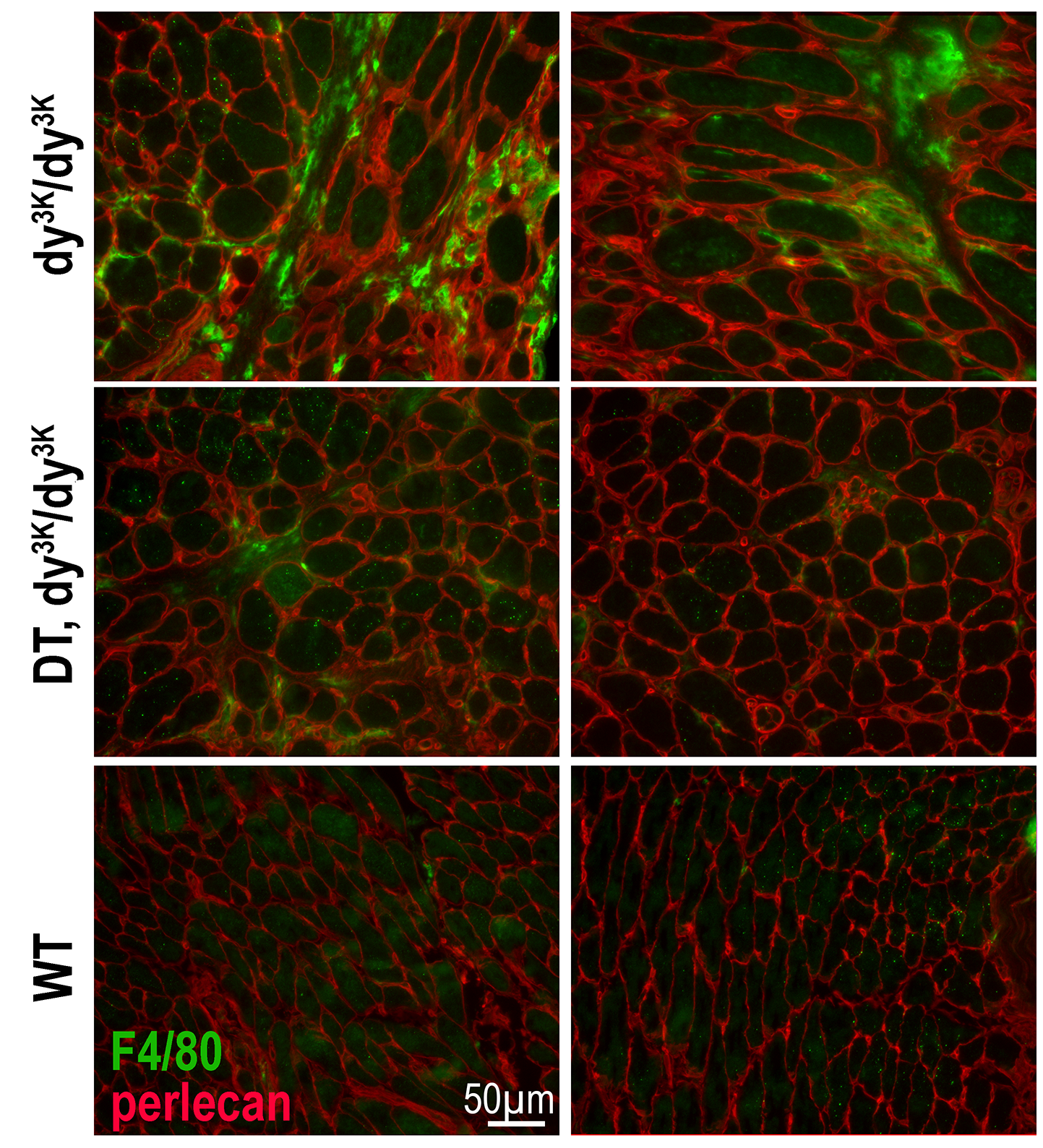


**Supplemental Figure S7.** *Inflammation is reduced in dy^3K^/dy^3K^ mice expressing αLNNd and mag.* Representative distal hindlimb muscle immunostained images are shown from 3-week-old mice stained for F4/80 (green), a macrophage marker, and BM perlecan (red).


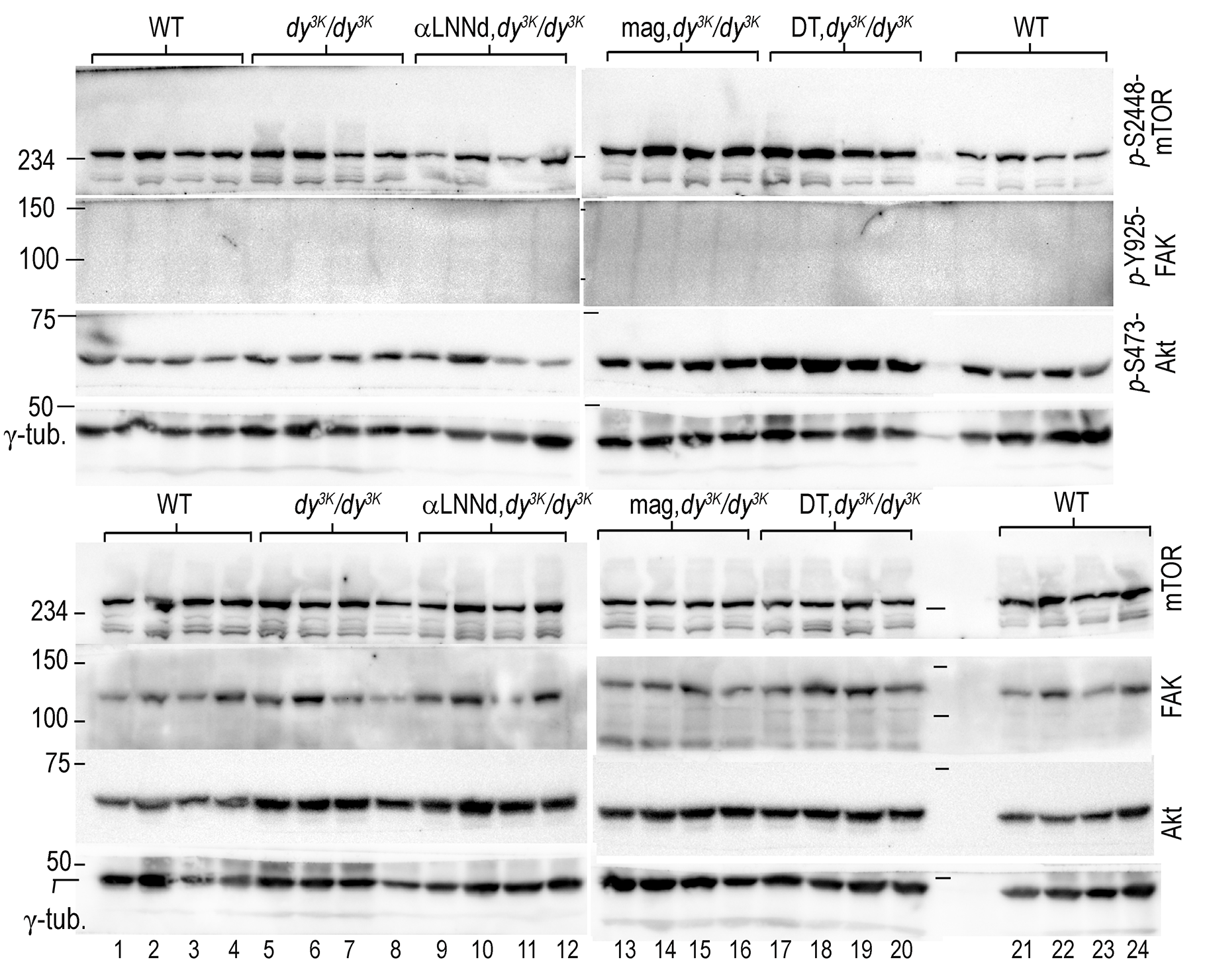


**Supplemental Figure S8.** *Muscle mTOR and Akt phosphorylation immunoblots.* Triceps muscle from 3-week-old mice (from four mice/condition) was ground and extracted with Triton buffer, subjected to reducing SDS-PAGE (7.5% gels), trans-blotted, and probed with rabbit anti-mTOR, anti-Akt, anti-FAK, as well as rabbit anti-phosphorylated mTOR, anti-phospho-Akt, and anti-phosphorylated FAK compared to loading control mouse anti-γ-tubulin. Phospho-mTOR and Akt bands were increased as a ratio over γ-tubulin in *mag*-*dy^3K^/dy^3K^* and DT-*dy^3K^/dy^3K^* muscle.


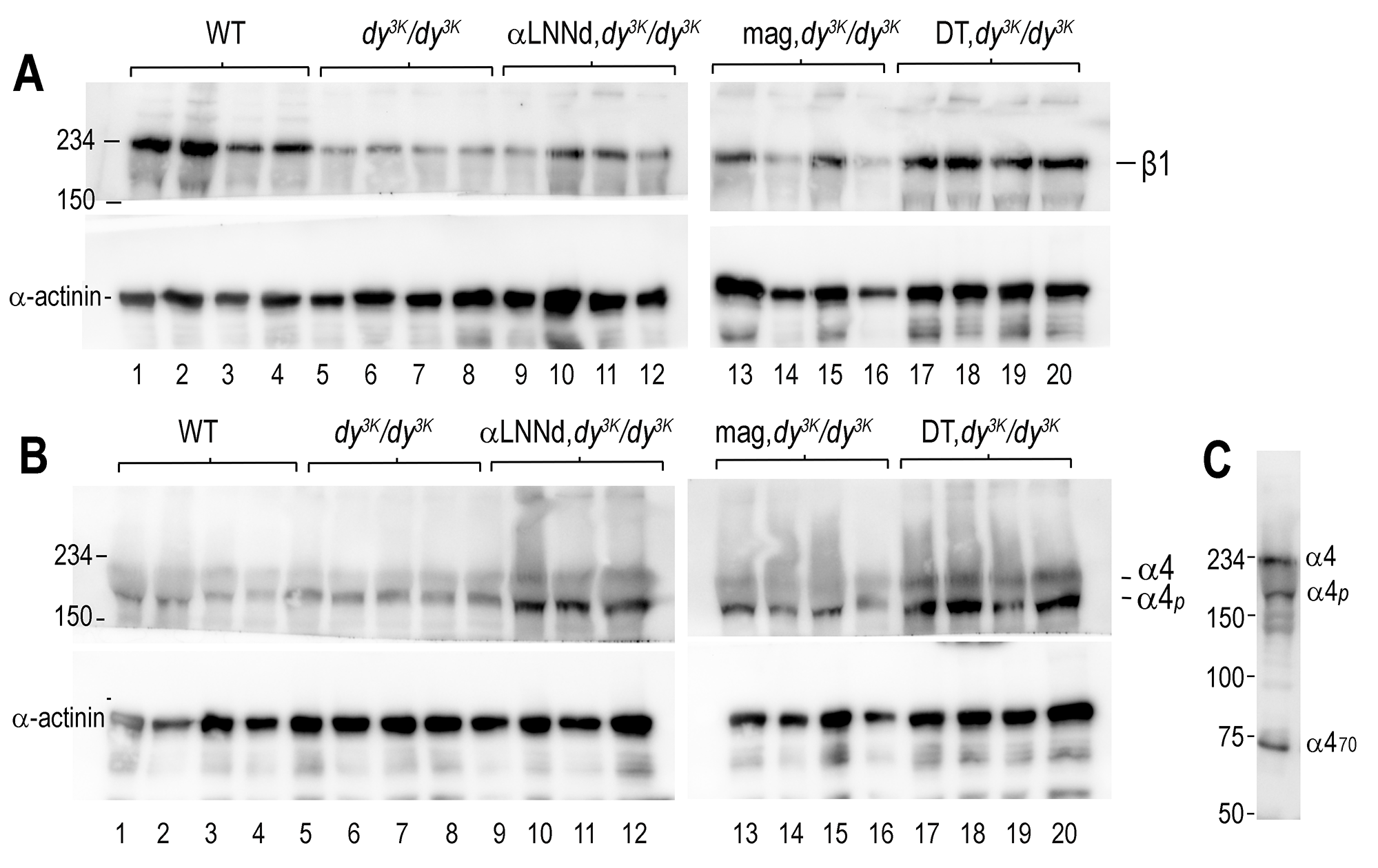


**Supplemental Figure S9.** *Immunoblots of laminin subunits extracted from muscle.* Triceps muscle, excised from 3-week-old WT, *dy^3K^/dy^3K^* and *dy^3K^/dy^3K^*-transgene mice (4 animals each condition), was ground, washed with Triton x-100 buffer to remove non-BM laminins, followed by SDS buffer extraction at 4^o^C. The SDS extracts were electrophoresed by SDS-PAGE (6 % gels, reducing conditions), trans-blotted, and probed with either rat mAb anti-Lmβ1 (Panel A) or chick polyclonal anti-Lmα4 (Panel B) antibodies and mouse mAb anti-α-actinin as a loading control. Panel C shows the electrophoretic migration of intact (α4) and processed (α4p + α470) Lmα4 subunit detected with specific antibody.

**Supplemental Figure S10.** *Muscle-extracted laminin sandwich-ELISA assays.* Hindlimb muscle from four mice for each condition (WT *dy^3K^/+*, dystrophic *dy^3K^/dy^3K^*, and double-transgene (DT) *dy^3K^/dy^3K^* mice extraction NP40 (non-matrix) and CEP (matrix) fractions and analyzed in sandwich ELISA assays to estimate laminin concentrations as described in the methods. The calculated values are shown in bar graphs (Fig. 7).


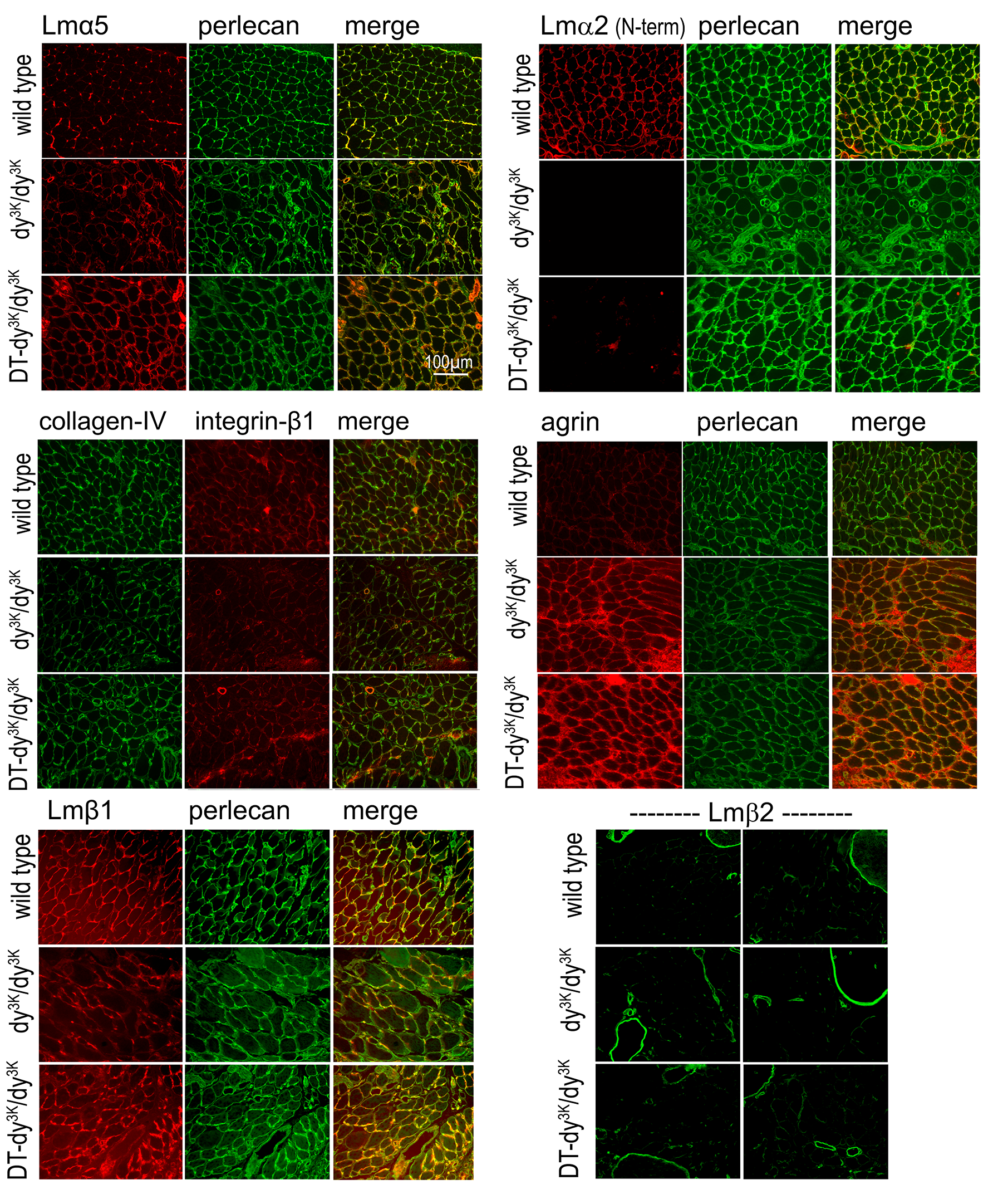


**Supplemental Figure S11**. *Basement membrane components*. Proximal hindlimb muscle from 3-week-old mice were immunostained with antibodies to detect BM laminins, perlecan, collagen-IV, and agrin. Laminin-α5 was confined to microvasculature in WT and detected in sarcolemma as well in *dy^3K^/dy^3K^* and DT- *dy^3K^/dy^3K^*. The sandwich assays indicate it is a minor laminin species in all conditions. Laminin-α2 (N-terminal monoclonal) was detected in WT but not dystrophic muscle. Collagen-IV was reduced in *dy^3K^/dy^3K^* and increased in DT- *dy^3K^/dy^3K^.* Endogenous (mouse) agrin was increased in sarcolemmal and microvascular *dy^3K^/dy^3K^* and DT- *dy^3K^/dy^3K^* relative to WT. Laminin-β1 was reduced in *dy^3K^/dy^3K^* and increased in DT-*dy^3K^/dy^3K^*. Laminin-β2, strongly present in perineurium, was only weakly present in muscle sarcolemma.


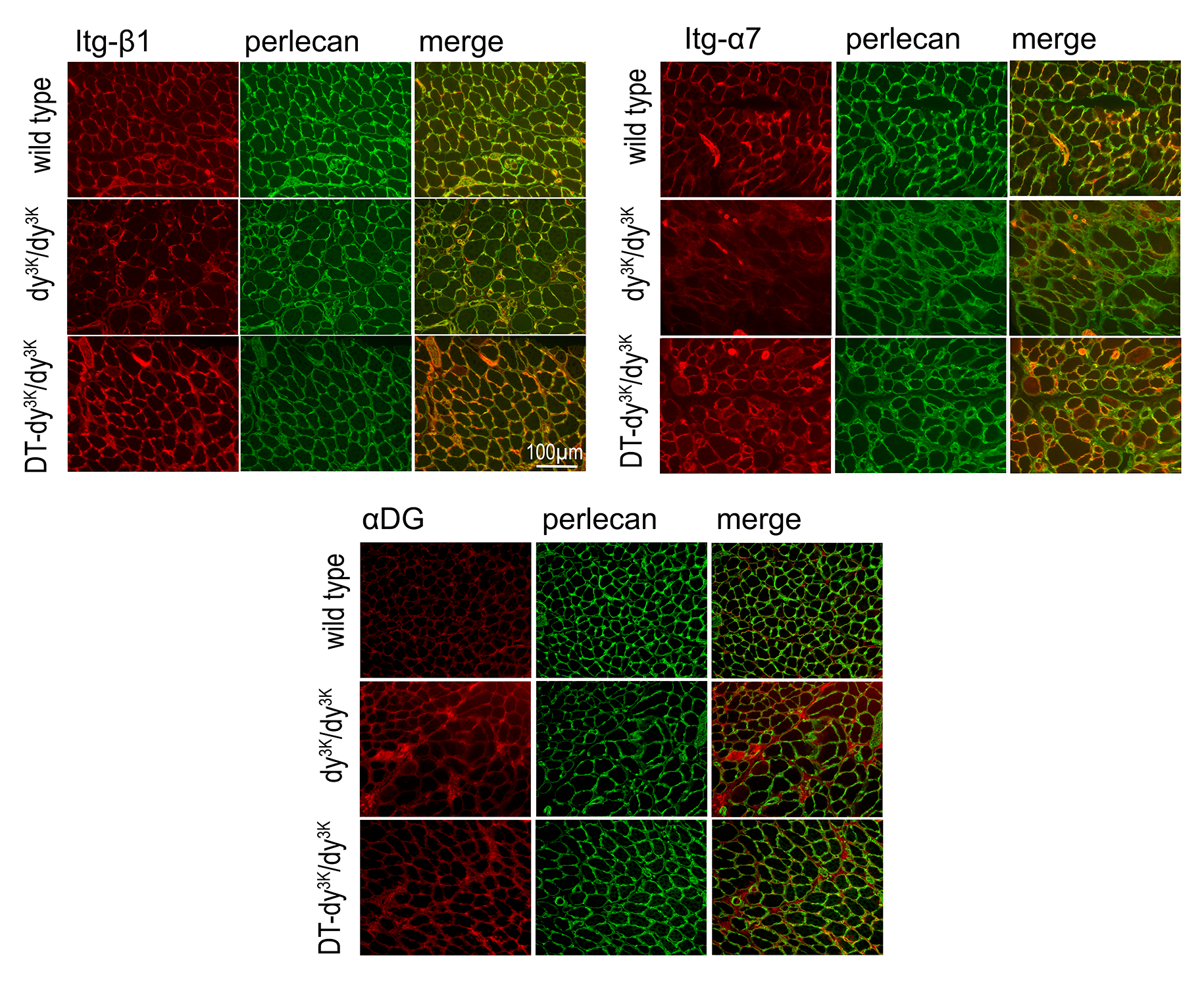


**Supplemental Figure S12**. *Basement membrane receptors.* Proximal hindlimb muscle from 3-week-old mice were immunostained with antibodies to detect BM β1-integrin, α7-integrin, and α-dystroglycan (αDG) (counterstained with perlecan antibody). Sarcolemmal β1- and α7-integrins were reduced in *dy^3K^/dy^3K^* and increased in DT- *dy^3K^/dy^3K^* relative to WT. Sarcolemmal α-dystroglycan (αDG) was increased in *dy^3K^/dy^3K^* and DT- *dy^3K^/dy^3K^* relative to WT.


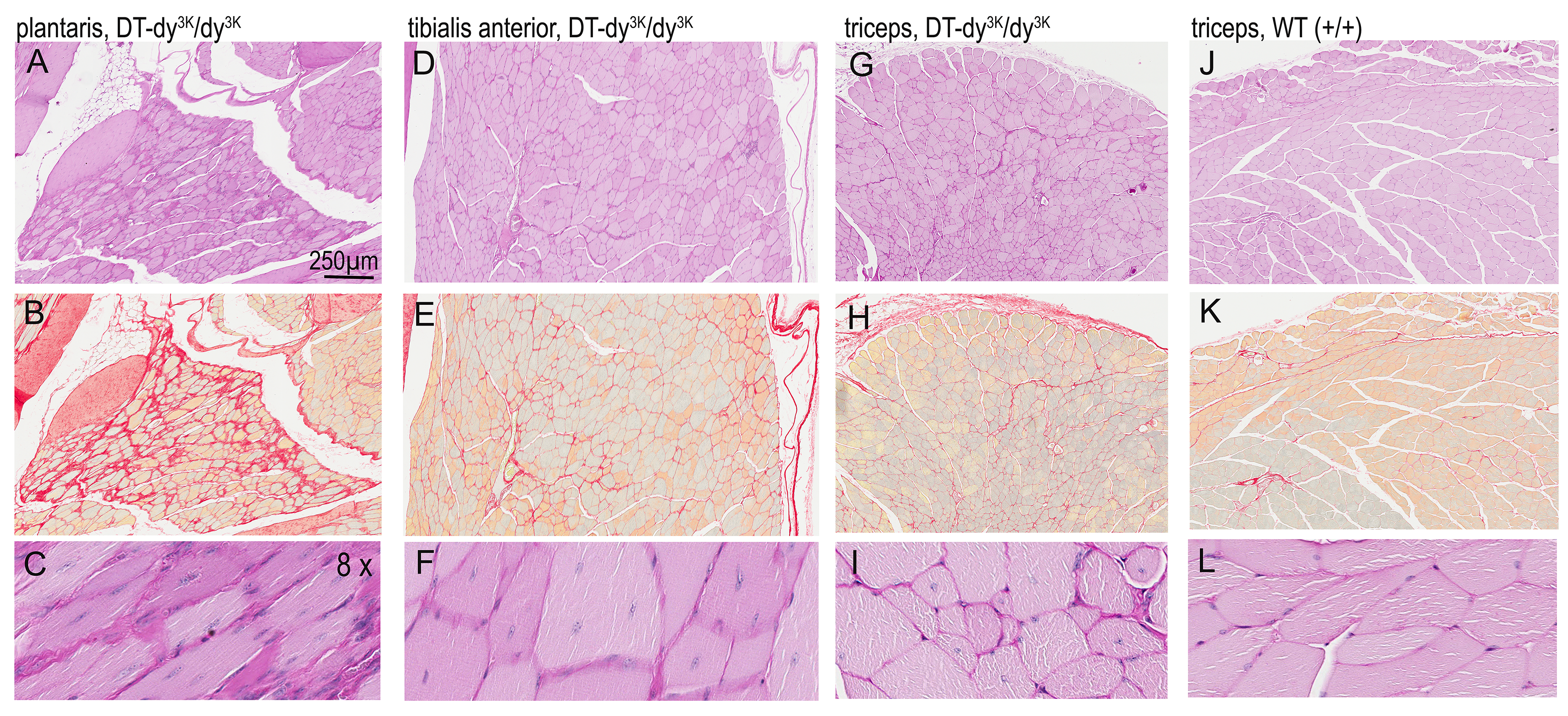


**Supplemental Figure S13**. *Histopathology of DT-dy^3K^/dy^3K^ WT muscle at one-year of age.* DT-*dy^3K^/dy^3K^* PAS (A,C, D, F, G, I) and picro Sirius red (B, E, H) for plantaris (A-C), tibialis anterior (D-F) and triceps (G-I) shown. Frequent central nuclei are noted for plantaris (32%, 4229/1354 myofibers), tibialis anterior (870/2335 myofibers) and triceps (37%, 1354/4229 myofibers). Tibialis anterior and triceps show little fibrosis, unlike plantaris. WT triceps (J-L) from a littermate is shown for comparison (3.2% central nuclei; 123/3788 myofibers).
